# A systemic pan-cancer analysis of MPZL3 as a potential prognostic biomarker and its correlation with immune infiltration and drug sensitivity in breast cancer

**DOI:** 10.3389/fonc.2022.901728

**Published:** 2022-07-29

**Authors:** Renhong Huang, Liangqiang Li, Zheng Wang, Kunwei Shen

**Affiliations:** ^1^ Department of General Surgery, Comprehensive Breast Health Center, Ruijin Hospital, Shanghai Jiao Tong University School of Medicine, Shanghai, China; ^2^ Department of Breast Surgery, Quanzhou First Hospital Affiliated to Fujian Medical University, Quanzhou, China

**Keywords:** MPZL3, biomarker, immune infiltration, drug susceptibility, prognosis, breast cancer

## Abstract

**Background:**

This study aimed to analyze the role of myelin protein zero-like 3 (MPZL3), a single membrane glycoprotein, in prognosis, tumor immune infiltration, and drug susceptibility in human cancers.

**Methods:**

Data regarding MPZL3 were extracted from the TCGA, GTEx, CellMiner, CCLE, TIMER, GSEA, and USCS Xena databases. The expression difference, survival outcomes, DNA methylation, tumor mutation burden (TMB), microsatellite instability (MSI), mismatch repair (MMR), tumor microenvironment (TME), immune cell infiltration, and drug sensitivity of MPZL3 were analyzed by R language software. Cell proliferation and drug sensitivity tests were applied to analyze the biological role of MPZL3 and drug sensitivities in breast cancer.

**Results:**

MPZL3 was highly expressed in most cancer types and correlated with unfavorable survival outcomes in several cancers. TMB, MSI, MMR, DNA methylation, and RNA modification played a significant role in mediating MPZL3 dysregulation in cancers, and MPZL3 was closely linked to CD8+ T cells and CD4+ T immune infiltration. The MPML3 mRNA level was associated with protein secretion, the Notch signaling pathway, and heme metabolism. In addition, drug sensitivity analysis and validation also indicated that MPZL3 expression influenced the sensitivity of therapeutics targeting EGFR, ABL, FGFR, etc. Additionally, MPZL3 overexpression contributed to proliferation and drug sensitivity in different subtypes of breast cancer.

**Conclusions:**

This study provides a comprehensive analysis and understanding of the oncogenic roles of the pan-cancer gene MPZL3 across different tumors, including breast cancer. MPZL3 could be a potential prognostic biomarker and therapeutic target for breast cancer.

## Introduction

Cancer has gradually become the most prominent cause of health-related death and remains a tricky problem that elicits tremendous burdens for both individuals and society (1). Strategies such as surgery, chemotherapy, radiotherapy, etc., have achieved certain results in cancer treatment, but a considerable number of patients with advanced disease still exhibit a poor prognosis. Recent advances in sequencing technology and bioinformatics have provided an unprecedented amount of data on the profile of the immune ecosystem, thus dramatically uncovering more regulatory mechanisms of the immune microenvironment and facilitating tumor immunotherapy (2).

The myelin protein zero-like 3 (MPZL3) gene encodes a single transmembrane protein that presents an immunoglobulin (Ig)-like variable (V)-type domain that may be involved in various cellular processes, such as cell adhesion, cell–cell interactions, and antigen binding (3). A previous study demonstrated that the MPZL3 protein can be considered a powerful intramitochondrial signaling hub that functions in circadian and metabolic regulation (4). Additionally, MZPL3 exerts a significant role in cell differentiation, lipid and energy metabolism, and immunity regulation (5). Previous genetic linkage studies have demonstrated that the MPZL3 chromosomal location (11q23.3) is closely linked to energy expenditure and body mass (6, 7). MPZL3 is capable of interacting with cellular proteins and activating intracellular signaling pathways (5). Moreover, MZPL3 contains immunoglobulin domain cell adhesion molecules that apply as regulators of immune cell recruitment during inflammation. MPZL3 might potentially function in the inflammatory response to dietary fat intake (8). The crosstalk between cancer cells and the surrounding tumor microenvironment (TME) regulates the development and tumorigenesis of cancer cells, thus impacting tumor apoptosis, invasion, metastasis, and therapeutic effects across cancers (9). In the TME, various components consist of the vasculature, collagen, fibroblasts, adipocytes, immune cells, myeloid-derived suppressor cells, bone marrow-derived cell (BMDC) signaling molecules, and the extracellular matrix (ECM) surrounding the tumor cells. Currently, immunotherapies, represented by immunological checkpoint blockade (ICB) and programmed death-1/ligand 1 (PD-1/L1), can be applied to unleash the antitumor immune response and achieve astounding therapeutic efficiency for a certain percentage of cancer patients (10). However, the roles and underlying mechanisms of MPZL3 in prognostic value, tumor immune infiltration, and drug susceptibility largely remain unclear.

In the present study, with the help of public databases such as The Cancer Genome Atlas (TCGA), we comprehensively investigated the relationship between MPZL3 expression and prognosis in different cancer types for the first time. We next analyzed the association of MPZL3 expression with immune cell infiltration and drug susceptibility in human cancers. Furthermore, we validated the role of MPZL3 in cell proliferation and drug sensitivity in different molecular subtypes of breast cancer cells.

## Methods and materials

### Data collection and preprocessing

Normalized expression profile data, TMB data, MSI data, and pan-cancer clinical information, including clear cell renal carcinoma, were downloaded from the UCSC Xena database (11, 12). For datasets in the USCS Xena databases, institutional review board approval and informed consent were not needed. Prognosis outcomes of MPZL3 in the TCGA Cohort were performed. Patients were excluded if they did not have prognostic information or died within 30 days. The survival information of pan-cancer including overall survival (OS), progression-free interval (PFI), disease-free interval (DFI), and disease-free survival (DSS) was extracted from the TCGA database to evaluate the prognostic value of MPZL3. OS was defined as the time from the date of diagnosis to the date of death due to any cause; DFI was defined as the interval from the date of the end of the initial treatment and the date at diagnosis of the recurrence; PFI was defined as the time between the date of diagnosis and the date of the first detection of progression or loss of follow-up; DSS was defined as the time from the clinical diagnosis to cancer-related death. Analysis of MPZL3 expression across cancers was performed in the TCGA database using p value <0.05 and absolute fold change >1.5 as the threshold. The abbreviations used in the study were as follows: ACC, adrenocortical carcinoma; BLCA, bladder urothelial carcinoma; BRCA, breast invasive carcinoma; CESC, cervical squamous cell carcinoma; CHOL, cholangiocarcinoma; COAD, colon adenocarcinoma; COADREAD, colorectal adenocarcinoma; DLBC, lymphoid neoplasm diffuse large B cell lymphoma; ESCA, esophageal carcinoma; GBM, glioblastoma multiforme; GBMLGG, lower-grade glioma and glioblastoma; LGG, lower grade glioma; HNSC, head and neck squamous cell carcinoma; KICH, kidney chromophobe; KIPAN, Pan-kidney cohort (KICH+KIRC+KIRP); KIRC, kidney renal clear cell carcinoma; KIRP, kidney renal papillary cell carcinoma; LAML, acute myeloid leukemia; LIHC, liver hepatocellular carcinoma; LUAD, lung adenocarcinoma; LUSC, lung squamous cell carcinoma; MESO, mesothelioma; OV, ovarian serous cystadenocarcinoma; PAAD, pancreatic adenocarcinoma; PCPG, pheochromocytoma and paraganglioma; PRAD, prostate adenocarcinoma; READ, rectum adenocarcinoma; SARC, sarcoma; SKCM, skin cutaneous melanoma; STAD, stomach adenocarcinoma; STES, stomach and esophageal carcinoma; TGCT, testicular germ cell tumors; THCA, thyroid carcinoma; THYM, thymoma; UCEC, uterine corpus endometrial carcinoma; UCS, uterine carcinosarcoma; and UVM, uveal melanoma.

### Enrichment analysis of MPZL3

Based on the guilt of association of a single gene in the expression profile, Pearson’s correlation between MPZL3 and other mRNAs retrieved from TCGA transcriptome data was analyzed. Sorted by the level of association index between genes and MPZL3, those genes most related to MPZL3 expression were selected for enrichment analysis. The R package “cluster profile” was used to perform Gene Set Enrichment Analysis (GSEA) (13).

### Assessment of potential chemotherapy drugs for MPZL3 expression

Clinical characteristics, including tumor stage and drug sensitivity, were introduced, and the relationship between MPZL3 expression and those characteristics was analyzed. The data, including IC50 (half maximal inhibitory concentration) and gene expression of cancer cell lines, were downloaded from the CellMiner database (https://discover.nci.nih.gov/cellminer/home.do) and GDSC (https://www.cancerrxgene.org/) database, respectively (14, 15).

### Differences in the tumor microenvironment and immunotherapy response

The R package “ESTIMATE” was introduced to evaluate the relationship between the infiltration degree of immune and stromal cells and MPZL3 expression across cancers. Coexpression analysis of immune-related genes and MPZL3 was performed *via* the R packages “ggpubr” and “ggcor”. The R package “CIBERSORT” was used to quantify the immune cell infiltration scores across cancers, and then the correlation of the degree of immune cells and MPZL3 expression was calculated. In addition, the correlation between neoantigen count, TMB, MSI and the expression of T-cell exhaustion marker genes (including PDCD1, TIGIT, CD274, CTLA4, LAG3, CXCL13, LAYN, and HAVCR2), DNA mismatch repair system genes (including MLH1, MSH2, MSH6, PMS2, and EPCAM), DNA methyltransferase (including DNMT1, DNMT2, DNMT3A, and DNMT3) and ESTIMATE scores and MPZL3 expression was analyzed. We also calculated the immune infiltration scores *via* the ssGSEA algorithm and analyzed the correlation and difference between immune cell infiltration and MPZL3 expression levels in BRCA. The TIMER website (http://timer.cistrome.org/) was used to validate the influence of MPZL3 mutation on immune cell infiltration in BRCA (16).

### Establishment of stably transfected cell lines

The overexpression vectors for human MPZL3 were constructed by Bioegene Co., Ltd. Briefly, the MPZL3 construct was generated by PCR‐amplified MPZL3 cDNA into a lentiviral plasmid with a puromycin-resistant gene. Subsequently, the collected lentiviral supernatants were applied to infect breast cancer cells, including MCF7, SKBR3, and MDA-MB-231 for stably transfected cell lines after puromycin screening with a concentration of 1 ug/mL for 72 hours.

### Western blot

Proteins were extracted using RIPA solution and then quantified using a BCA protein assay kit (Pierce, 23227). The proteins were separated by 12% SDS polyacrylamide gels and transferred onto polyvinylidene difluoride (PVDF) membranes. Subsequently, the membranes were blocked using a 5% skimmed milk solution for 1 h at 25°C. Next, the membranes were incubated with primary antibodies against GAPDH (1:1000, Abcam, ab8245) or MPZL3 (1:1000, Abcam, ab76327) at 4°C overnight. Finally, the membranes were incubated with a secondary antibody and imaged using an enhanced chemiluminescence (ECL) detection system (Thermo Scientific, USA).

### Proliferation and clone-formation assays

For the proliferation assay, 3×10^3^ cells suspended in 100 µl of DMEM were seeded onto 96-well plates. Cell proliferation was assessed using the CellTiter-Glo assay (Promega, USA). For the clone-formation assay, 700 cells were seeded onto 6-well plates and incubated at 37°C for 2 weeks. Cells were fixed with 4% paraformaldehyde and stained with 0.2% crystal violet for 20 min. Cells were washed with PBS and imaged, and the clones were counted.

### Drug sensitivity detection in different subtypes of breast cancer cells

To analyze the effect of MPZL3 expression on drug sensitivity. We selected the ER-positive cell line MCF7, the ERBB2-amplified cell line SKBR3, and the triple-negative breast cancer cell line (TNBC) MDA-MB-231 to detect fulvestrant, pyrotinib, and paclitaxel sensitivity after transfection with the MPZL3-overexpression plasmid. Briefly, cells were seeded onto 96-well plates and treated with different concentrations of drugs. After 5 days of incubation, cell viability was measured using the CellTiter-Glo assay, and the IC50 of each drug was calculated.

### Statistical analysis

Differences in the expression of MPZL3 in the public datasets were compared by one-way ANOVA, and differences in clinical information and immune checkpoint inhibitor response between the two different subgroups were compared by the chi-squared test. Differences in OS and PFI between the two subgroups were compared by the Kaplan–Meier method and log-rank rest. The hazard ratios (HRs) were calculated by univariate Cox regression and multiple Cox regression analysis. All image analyses in this study were performed using ImageJ software. All P values were two-sided, with P value less than 0.05 considered significant. The adjusted P value was obtained by Benjamini–Hochberg (BH) multiple test correction. All data processing, statistical analysis, and plotting were conducted using R 4.0.4 software.

## Results

### MZPL3 expression analysis between tumor and normal tissue samples

**Figure 1:**
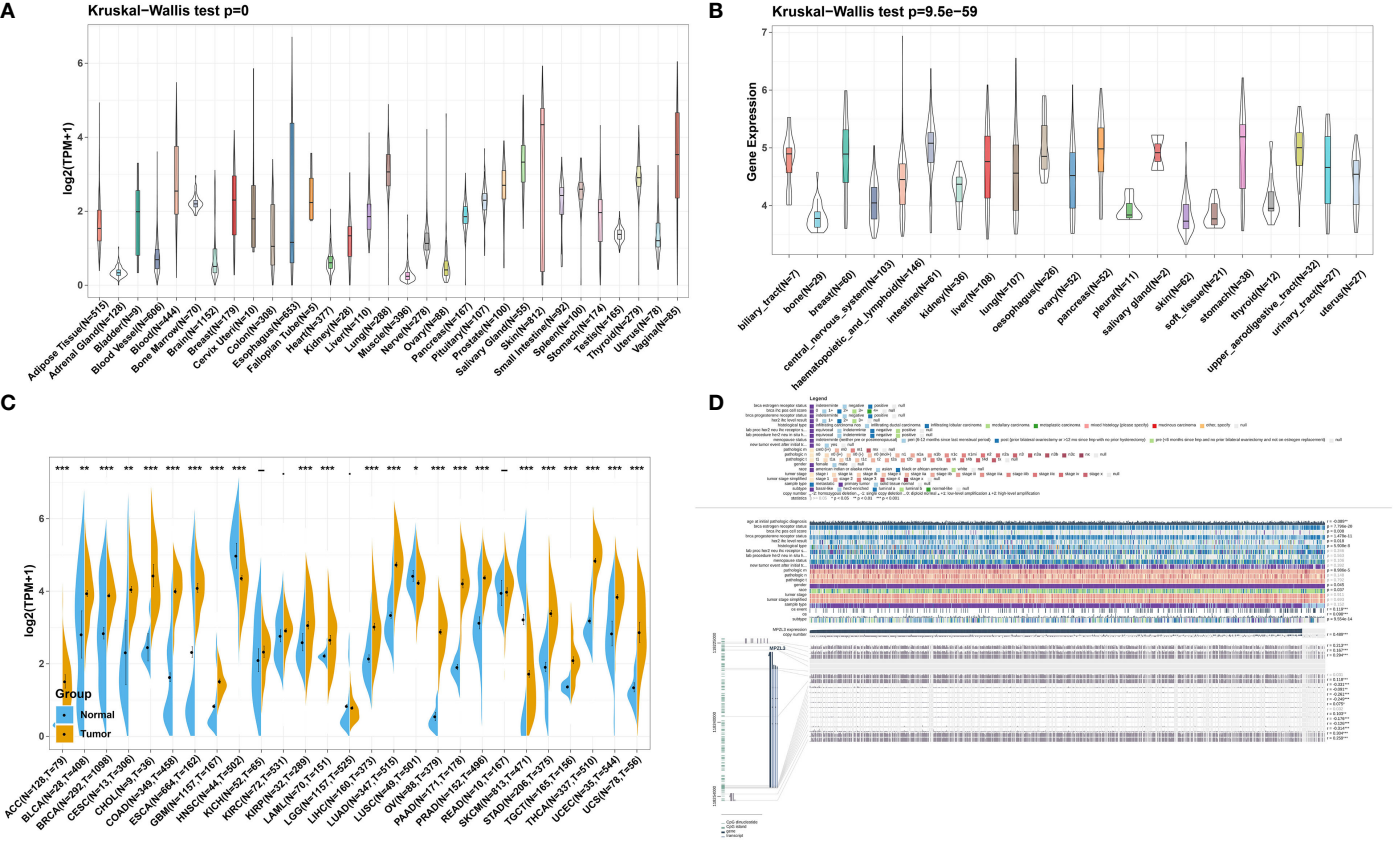
MPZL3 expression level in pan-cancer. **(A)** Comparison of MPZL3 expression in normal tissues in the GTEx database. **(B)** MPZL3 expression differed in tumor cell lines from the CCLE database. **(C)**. MPZL3 expression differed in paired tumor and normal tissues of 27 cancer types from the TCGA and GTEx databases. **(D)** Correlations between the MPZL3 level and clinicopathological characteristics in BRCA. MPZL3, Myelin Protein Zero-like 3; GTEx, Genotype-Tissue Expression; CCLE, Cancer Cell Line Encyclopedia; TCGA, Cancer Genome Atlas; BRCA, breast invasive carcinoma. (*p < 0.05, **p < 0.01, ***p < 0.001).

We first analyzed the physiological MPZL3 gene expression levels of 31 tissues across tissues using the GTEx database, and the results are indicated in **Figure 1A**. The results indicated that MPZL3 was highly expressed in skin, vagina, salivary gland, and lung tissues. In addition, relative MPZL3 expression levels across 21 cell lines from CCLE data are depicted in **Figure 1B**. We found that MPZL3 expression was different in paired tumor and normal tissues of 27 cancer types based on the data from the TCGA and GTEx databases. Compared with normal tissues, MPZL3 expression was remarkably higher in ACC, BLCA, BRCA, CESC, CHOL, COAD, ESCA, GBM, KIRP, LAML, LIHC, LUAD, OV, PAAD, PRAD, STAD, TGCT, THCA, UCEC and UCS and was expressed at lower levels in HNSC, LUSC, and SKCM (**Figure 1C**). Thus, the data demonstrated that MPZL3 is abnormally expressed in different cancers. Furthermore, we focused on MPZL3 expression in BRCA and found that the MPZL3 expression copy number correlated with clinicopathological characteristics such as BRCA IHC positive status, HER2 IHC level, histological type, sex, race, and molecular subtype in BRCA from the MEXPRESS database (**Figure 1D**).

### Correlation analysis of MPZL3 expression with genetic alterations

**Figure 2:**
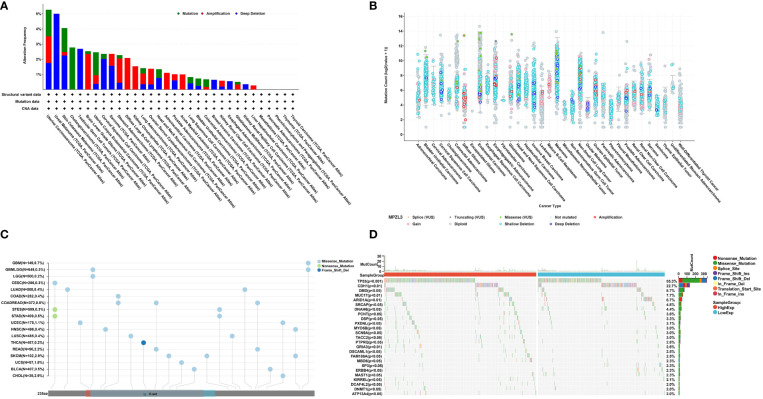
MPZL3 mutation across cancers. **(A, B)** The mutation frequency and mutation count of MPZL3 in pan-cancer by the cBioPortal database.**(C)** Mutation diagram of MPZL3 in different cancer types across protein domains. **(D)** The different mutation landscapes in the MPZL3 high- and low-expression groups in BRCA. MPZL3, myelin protein zero-like 3; BRCA, breast invasive carcinoma.

We further assessed the mutation frequency and mutation count, including mutation, amplification, and deep deletion of MPZL3, in different tumor samples by the cBioPortal database. As shown in **Figure 2A**, the highest alteration frequency of MPZL3 (alteration frequency > 3%) appeared in UCEC patients with “mutations” as the primary type, and the “deep deletion” mutation of MPZL3 was the main mutation type in uveal melanoma. The “amplification” type of CNA is the primary type in brain lower grade glioma and diffuse-large B-cell lymphoma cases, with an alteration frequency of approximately 2% to 3% (**Figures 2A, B**). The MPZL3 genetic alteration in different cancer types across protein domains was also detected, and missense mutation of MPZL3 was the main type of genetic alteration (**Figure 2C**). We also assessed the mutation landscape in the MPZL3 high- and low-expression groups in BRCA. Whether in the MPZL3 high- or low-expression group, TP53, CDH1, DMD, MUC17, and ARID1A were the top five mutated genes, with a frequency of more than 5%. Compared with the cohort with low MPZL3 expression, the cohort with high MPZL3 expression had a higher level of TP53 mutation and a lower level of CDH1 mutation (**Figure 2D**).

### MZPL3 expression is related to DNA methylation and RNA modification

**Figure 3:**
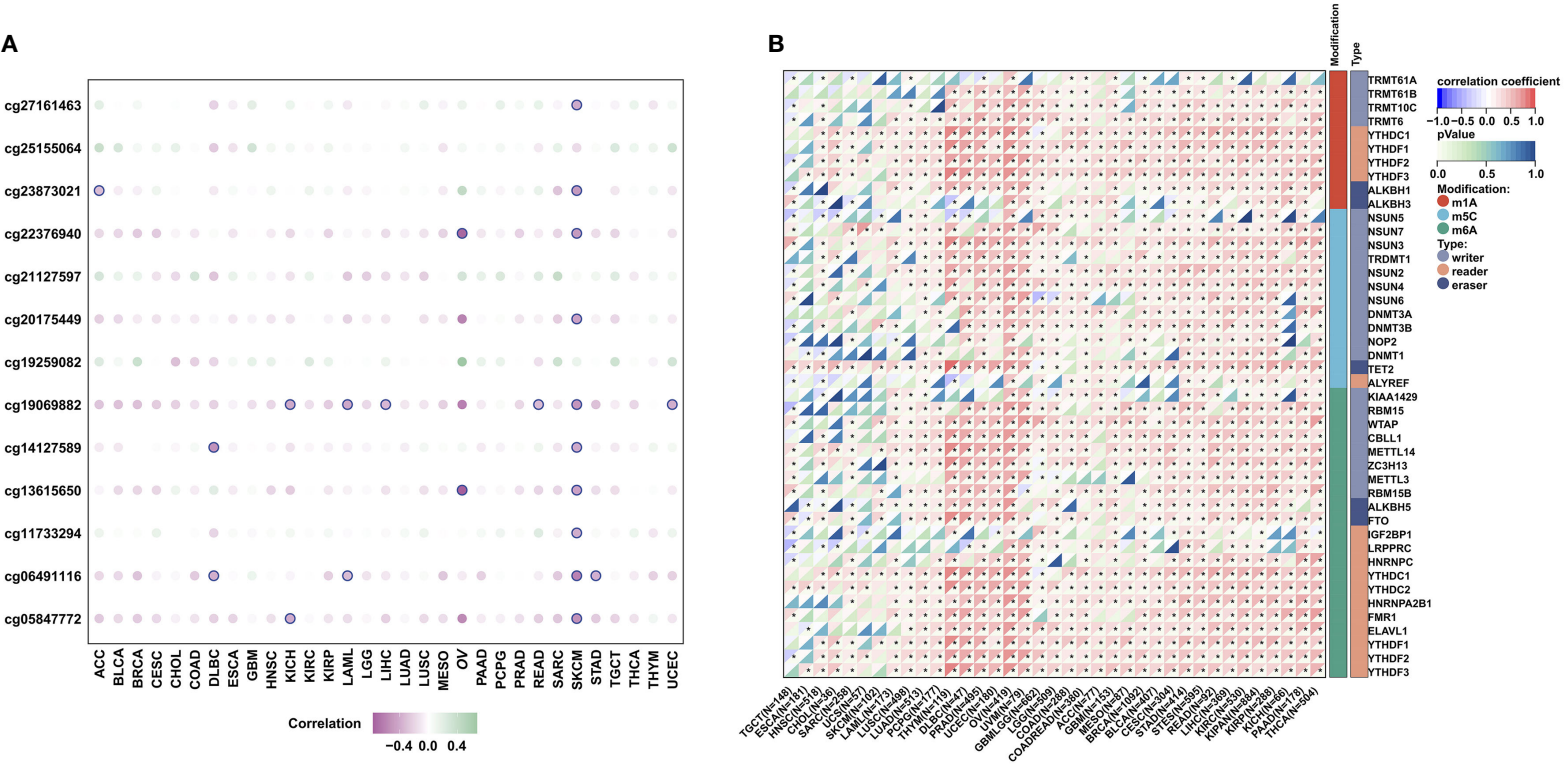
DNA methylation and RNA modification in MPZL3. **(A)** The correlation of MPZL3 expression and the methylation degree across cancers. **(B)** The correlation of MPZL3 expression and RNA modification regulator expression across cancers. MPZL3, Myelin Protein Zero-like 3. (*p<0.05).

To further analyze the potential regulatory effect of DNA methylation and RNA modification on MPZL3 expression, we first systematically explored the correlation of the DNA methylation level and MPZL3 expression, which indicated that the majority of the CG sites of DNA methylation could negatively regulate MPZL3 expression (**Figure 3A**). In addition, RNA modification-related genes (including m1A, m5C, and m6A) were also significantly positively correlated with MPZL3 expression (**Figure 3B**). All of these results indicated that MPZL3 expression might perform its regulatory function mainly *via* posttranscriptional modification.

### High MZPL3 expression predicts unfavorable survival outcomes

**Figure 4:**
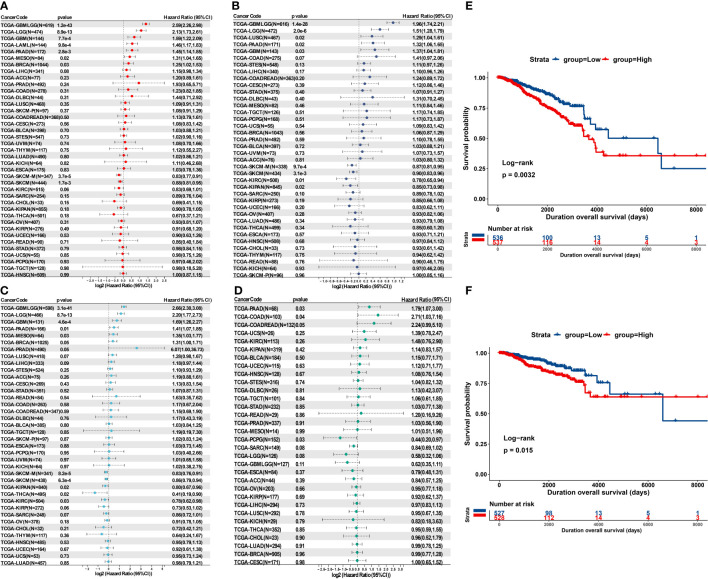
Prognostic value of MPZL3 in cancers. Relationship between MPZL3 expression and prognosis, including OS **(A)**, PFI **(B)**, DSS **(C)**, and DFI **(D)**, across cancers from the TCGA database. Survival curves of OS **(E)** and DSS **(F)** in BRCA patients using the Kaplan-Meier analysis. MPZL3, myelin protein zero-like 3; OS, overall survival; PFI, progression-free interval; DSS, disease-specific survival; DFI, disease-free interval (DFI); BRCA, breast invasive carcinoma; TCGA, The Cancer Genome Atlas.

To investigate the potential prognostic value of MPZL3 in cancers, we integrated the MPZL3 mRNA expression level with the overall survival (OS), progression-free interval (PFI), disease-specific survival (DSS) and disease-free survival (DFI) of the 33 cancer types in the TCGA database. Then, Cox proportional hazards models and Kaplan–Meier survival analysis were employed to evaluate the prognostic potential of MPZL3 expression. We analyzed the correlation of MPZL3 expression with OS, PFI, DSS and DFI in type of cancers, and the results are displayed in forest charts. With regard to OS, patients with MPZL3 expression had a relatively worse OS in GBMLGG (HR=2.59, 95% CI 2.26 - 2.98, P<0.001), LGG (HR=2.13, 95% CI 1.73 - 2.61, P<0.001), GBM (HR=1.59, 95% CI 1.22 - 2.09, P<0.001), LAML (HR=1.46, 95% CI 1.17 - 1.83, P<0.001), PAAD (HR=1.45, 95% CI 1.14 - 1.85, P<0.01), MESO (HR=1.31, 95% CI 1.04 - 1.65, P=0.02) and BRCA (HR=1.25 95% CI 1.02 - 1.53, P=0.03) (**Figure 4A**), worse PFI in GBMLGG (HR=1.96, 95% CI 1.74 - 2.21, P<0.001), LGG (HR=1.51, 95% CI 1.28 - 1.79, P<0.001), LUSC (HR=1.29, 95% CI 1.04 - 1.61, P=0.02), PAAD (HR=1.32, 95% CI 1.06 - 1.65, P=0.02) and GBM (HR=1.37, 95% CI 1.04 - 1.81, P=0.03) (**Figure 4B**), worse DSS in GBMLGG (HR=2.66, 95% CI 2.30 -3.08, P<0.001), LGG (HR=2.20, 95% CI 1.77 - 2.73, P<0.001), GBM (HR=1.69, 95% CI 1.26 - 2.27, P<0.001), PAAD (HR=1.41, 95% CI 1.07 - 1.85, P=0.01), MESO (HR=1.35, 95% CI 1.03 - 1.77, P=0.03), BRCA (HR=1.31, 95% CI 1.00 - 1.71, P=0.05), and PRAD (HR=6.07, 95% CI 1.00 - 36.73, P=0.05) (**Figure 4C**), and worse DFI in PAAD (HR=1.79, 95% CI 1.07 - 3.00, P=0.03), COAD (HR=2.71, 95% CI 1.03 - 7.16, P=0.04), COADREAD (HR=2.24, 95% CI 0.99 - 5.10, P=0.05) (**Figure 4D**). Higher levels of MPZL3 mRNA were also linked with better OS in SKCM-M (HR=0.83, 95% CI 0.77 - 0.91, P<0.001) and SKCM (HR=0.88, 95% CI 0.81 - 0.95, P<0.01) (**Figure 4A**), better PFI in SKCM-M (HR=0.87, 95% CI 0.81 - 0.95, P<0.05), SKCM (HR=0.90, 95% CI 0.83 - 0.96, P<0.01), KIRC (HR=0.78, 95% CI 0.65 - 0.94, P=0.01) and KIPAN (HR=0.85, 95% CI 0.73 - 0.98, P=0.02) (**Figure 4B**), and better DSS in SKCM-M (HR=0.83, 95% CI 0.76 - 0.91, P<0.001), SKCM (HR=0.86, 95% CI 0.79 - 0.94, P<0.001), KIPAN (HR=0.80, 95% CI 0.67 - 0.96, P=0.02), THCA (HR=0.41, 95% CI 0.19 - 0.90, P=0.02) and KIRC (HR=0.78, 95% CI 0.62 - 0.98, P=0.03) (**Figure 4C**), and better DFI in PCPG (HR=0.44, 95% CI 0.20 - 0.97, P=0.03). Additionally, the Kaplan-Meier analysis results indicated that MPZL3 mRNA expression is significantly related to prognosis in cancers, especially patients with GBM, BRCA, LGG, and PAAD (**Figures 4E, F** and **S1**–**4**).

### Integrative analysis of the association between MPZL3 expression and hallmarks

**Figure 5:**
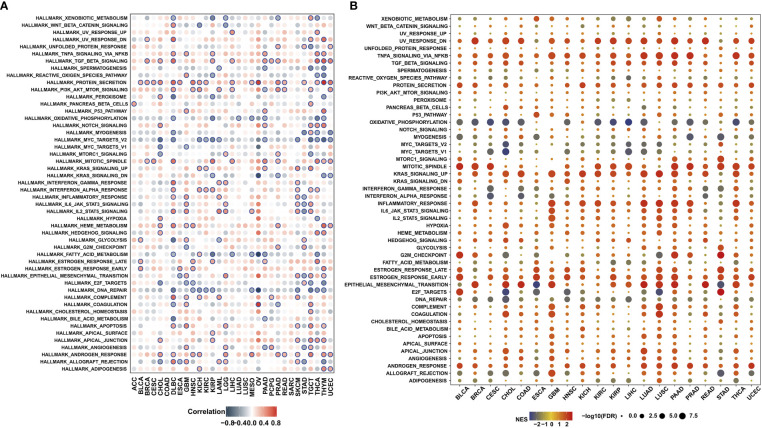
Association between MPZL3 and hallmarks across cancers. **(A)** Correlations of MPZL3 expression with HALLMARKS enrichment scores in 33 cancer types. **(B)** Enrichment analysis of hallmarks in the MPZL3 high- and low-expression groups. MPZL3, Myelin Protein Zero-like 3.

Furthermore, we conducted co-expression analyses to analyze the correlations of MPZL3 expression with HALLMARKS enrichment scores in 33 cancer types. The analyzed genes encoded tumor-associated signaling pathways, immune regulation, cell cycle, apoptosis, chemokine-related pathways, chemokine receptor proteins, etc., and the heatmap indicated that these hallmarks are closely associated with MPZL3 expression. Hallmarks such as TGF-β signaling, protein secretion, PI3K-AKT pathway, mitotic spindle, Kas signaling, IFN-α, IFN-γ, IL6-JAK-STAT3 signaling, IL2-STAT5 signaling, HEME metabolism, estrogen response, complement pathway, apoptosis pathway, androgen response were mostly positively correlated with MPZL3 expression in pan-cancers, while hallmarks such as xenobiotic metabolism, oxidative phosphorylation, Myc-targets, fatty-acid metabolism, E2F targets, DNA repair were mostly negative correlation with MPZL3 expression (**Figure 5A**). Furthermore, hallmarks in the MPZL3 high- and low-expression groups were identified by GSEA enrichment analysis. Hallmarks in the high MPZL3-expression groups were mainly enriched in UV-response upregulation, TNF-α signaling pathway, mitotic spindle, Kas signaling upregulation, inflammatory response, estrogen response, epithelial-mesenchymal transition (EMT), etc. The hallmarks of the low MPZL3-expression groups mostly correlated significantly with oxidative phosphorylation, myogenesis, Myc targets, DNA repair, etc. (**Figure 5B**). All of these results implied that MPZL3 expression actively participates in various biological processes and that different levels of MPZL3 mRNA exert different biological functions in different types of cancers.

### Association of MPZL3 expression with stemness index, TMB, MSI, MMR, and DNA methyltransferases

**Figure 6:**
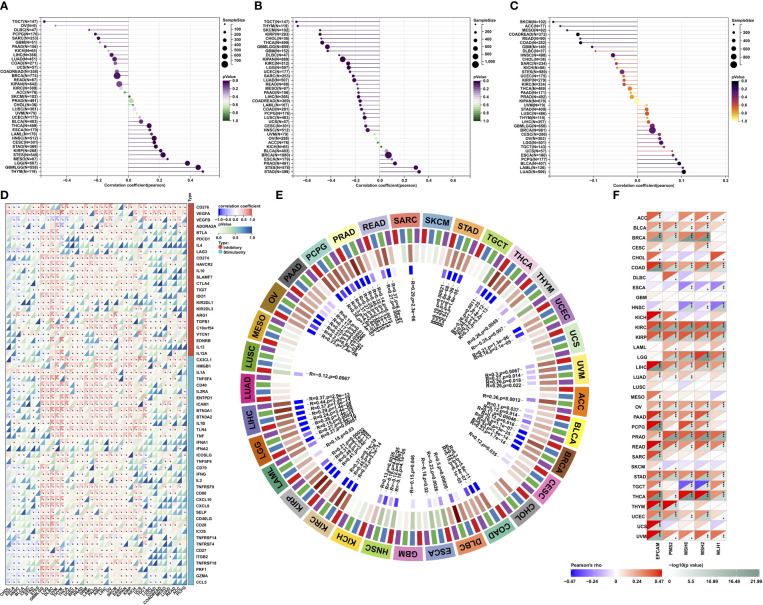
Correlations of MPZL3 with the stemness index, TMB, MSI, MMR, and DNA methyltransferases across cancers. **(A, B)** Correlations of MPZL3 expression with DNAss and RNAss index in 33 cancer types. **(C)** Correlations of MPZL3 expression with TML in 33 cancer types. **(D)** Spearman’s correlation analysis of MPZL3 expression with immune-regulated genes across cancers. **(E)** Spearman’s correlation analysis of MPZL3 expression with four DNA methyltransferases across cancers (red indicates DNMT1; blue indicates DNMT2; green indicates DNMT3A; purple indicates DNMT3B). **(F)** Spearman’s correlation analysis of MPZL3 expression with the expression levels of five MMR genes across cancers. MPZL3, myelin protein zero-like 3; TMB, tumor mutational burden; MSI, microsatellite instability; MMR, mismatch repair; DNMT, DNA methyltransferase. (*p < 0.05, **p < 0.01, ***p < 0.001).

We further investigated the possible roles of MPZL3 in the stemness of cancers. The intersected transcription of MPZL3 expression data with stemness scores (DNA methylation-based and RNA methylation-based) was determined using the Spearman correlation test. Notably, the majority of MPZL3 expression positively correlated with DNAss and RNAss in the 33 TCGA cancers, which suggested that high MPZL3 expression predicted a high index of the tumor stemness score (DNAss and RNAss), strong activity of tumor stem cells and a low degree of tumor differentiation (**Figures 6A, B**).

Tumor mutational burden (TMB), microsatellite instability (MSI), and mismatch repair (MMR) are independent biomarkers that complement each other to predict the efficacy and effect of immune therapeutics. MPZL3 expression was strongly positively or negatively associated with TMB in 33 types of cancers (**Figure 6C**). High MPZL3 expression correlated positively with TMB in BRCA, ESCA, LGG, PAAD, STAD, THYM, and BLCA and negatively with TMB in COAD, KIRC, SARC, and THCA (**Figure S5A**). In addition, high MPZL3 expression correlated positively with MSI in READ, STAD, and UCEC and negatively with MSI in DLBC, LUSC, SKCM, and UCS (**Figure S5B**).

We also analyzed the correlation between MPZL3 and the expression of 60 immune checkpoint genes, including immune inhibitory and stimulatory genes (**Figure 6D**). Interestingly, in GBMLGG, LGG, DLBC, UVM, THYM, THCA, UCEC, BRCA, PRAD, GBM, LAML, PAAD, KIRC, LIHC, OV, SKCM, SARC, KIPAN, KIRP, KICH, UCS, and TGCT, MPZL3 expression correlated with more than 50 immune checkpoint markers. All of these results indicated that MPZL3 played a significant role in tumor immunity regulation.

The association of MPZL3 expression with all four methyltransferases in 33 types of cancer was also evaluated (**Figure 6E**). In general, MPZL3 expression was highly associated with four DNA methyltransferases in the majority of cancers. In MESO, SKCM, UCS, CHOL, KICH, and LUAD, these cancers did not show any correlation with the four methyltransferases. To investigate the potential role of MPZL3 in tumor progression, the association of MZPL3 expression with mutation levels of five MMR genes, including EPCAM, PMS2, MSH6, MSH2, and MH1, was evaluated (**Figure 6F**). The results revealed that MPZL3 was highly related to MMR genes in 33 cancers, except for GBM and LUSC. These results imply that MPZL3 may be involved in the regulation of tumor progression by mediating DNA repair and DNA methylation across cancers.

### Correlation of MPZL3 expression with immune infiltration

**Figure 7:**
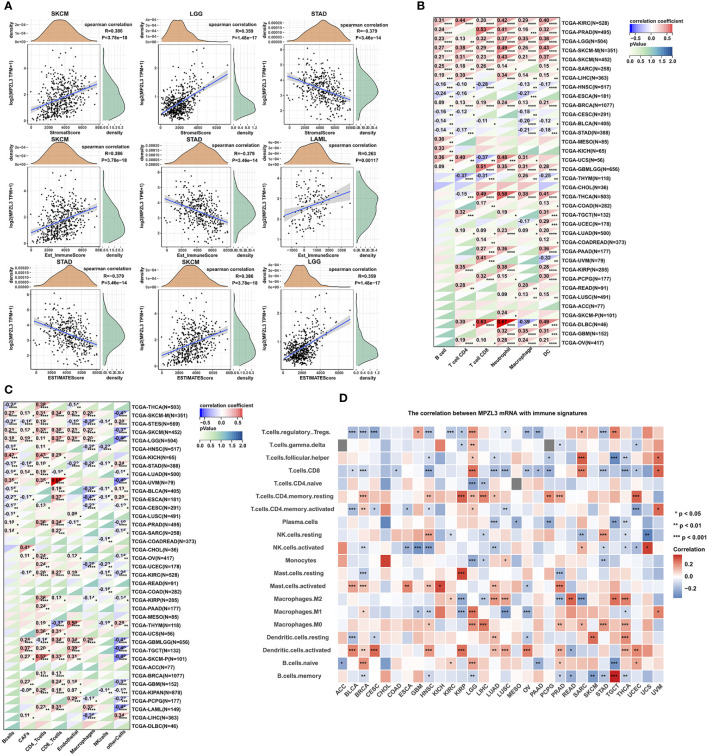
Correlation of MPZL3 expression with immune infiltration in various cancers. **(A)** Top three cancers by ImmuneScore, StromalScore, and ESTIMATEScore. **(B)** Spearman’s correlation analysis of MPZL3 expression with immune infiltration *via* timer algorithms. **(C)** Spearman’s correlation analysis of MPZL3 expression with immune infiltration *via* MCPcounter algorithms. **(D)** Spearman’s correlation analysis of MPZL3 expression with immune infiltration *via* CIBERSORT algorithms. MPZL3, Myelin Protein Zero-like 3. CIBERSORT, Cell-type Identification by Estimating Relative Subsets of RNA Transcripts. (*p < 0.05, **p < 0.01, ***p < 0.001).

Indeed, tumors are often infiltrated by various numbers of immune cells, such as lymphocytes, macrophages, and mast cells. To investigate the role of MPZL3 in tumor immune infiltration, we integrated the ImmuneScore, StromalScore, and ESTIMATEScore across cancers with MPZL3 expression. The results demonstrated that MPZL3 expression positively correlated with the ImmuneScore in DLBC, GBM, KIRC, etc., but negatively correlated with the ImmuneScore in BLCA, CESC, COAD, etc. (**Figure S7**). Similarly, in the StromalScore, MPZL3 expression positively correlated with MPZL3 expression in GBM, KIRC, LAML, etc., but negatively correlated with MPZL3 expression in BLCA, CESC, ESCA, etc. (**Figure S8**). Then, MPZL3 expression was also positively correlated with the ESTIMATEScore in DLBC, GBM, KIRC, etc., while it negatively correlated with the ESTIMATEScore in BLCA, CESC, ESCA, etc. (**Figure S9**). We calculated the top three tumors that were most significantly associated with MPZL3 expression, in which SKCM, LCG, and STAD were positively correlated with the StromalScore; SKCM, STAD, and LAML were negatively correlated with the ImmuneScore; and STAD, SKCM, and LGG were positively correlated with the ESTIMATEScore (**Figure 7A**). Next, we analyzed the scores of infiltrating immune cells in BRCA from the TIMER database and then investigated the correlation between the MPZL3 expression level and immune infiltration levels. MPZL3 expression was appreciably positively correlated with the infiltration levels of 6 immune cell types, including B cells, CD4+ T cells, CD8+ T cells, neutrophils, macrophages, and dendritic cells (**Figure S10**).

Next, we applied the TIMER, MCPcounter, and CIBERSORT algorithms to further investigate the potential relationship between the infiltration level of various immune cells and MPZL3 expression in different types of cancers from the TCGA database. In the algorithms of the TIMER algorithm, we analyzed the association of B cell, CD4+ T cell, CD8+ T cell, neutrophil, macrophage, and DC infiltration with MPZL3 expression. This result indicated that a positive correlation was observed between the association of these immune cell infiltrations and MPZL3 expression in KIRC, PRAD, LGG, SKCM, SARC, LIHC, GBML, BRCA, etc. (**Figure 7B**). In addition, a significant positive correlation was observed between the association of CD8+ T cells and CD4+T immune infiltrations with MPZL3 expression in most types of cancers based on MCPcounter algorithms (**Figure 7C**). The CIBERSORT algorithm also indicated that MPZL3 mRNA expression exhibited varying degrees of immune infiltration signatures in different types of cancers (**Figure 7D**). We also analyzed the association of MPZL3 somatic copy number alterations with immune infiltration levels in different types of BRCA. In BRCA, MPZL3 somatic copy number alterations of deep deletion and arm-level deletion were closely associated with immune infiltration of B cells, CD8+ T cells, CD4+ T cells, macrophages, neutrophils, and DCs. No signature correlation of MPZL3 somatic copy number alterations with immune infiltration was found in the basal and Her-2 subtypes of BRCA, while in the luminal subtype, MPZL3 somatic copy number alterations with deep deletion were associated with B-cell and CD4+ T-cell infiltration, arm-level deletion was associated with CD4+ T-cell infiltration, and arm-level gain was associated with CD8+ T cell, CD4+ T-cell and neutrophil infiltration (**Figure S6**).

### Functional enrichment of high and low MPZL3 expression

Functional enrichment analysis regarding high and low MPZL3 mRNA expression was performed using GSEA. KEGG enrichment analysis showed that high MPZL3 expression was mainly associated with renal cell carcinoma, the neurotrophin signaling pathway and the vasopressin-regulated water pathway. HALLMARK enrichment suggested that high MPZL3 mRNA expression was associated with protein secretion, the Notch signaling pathway, and heme metabolism (**Figure S11**).

### MPZL3 expression with different drug sensitivities

**Figure 8:**
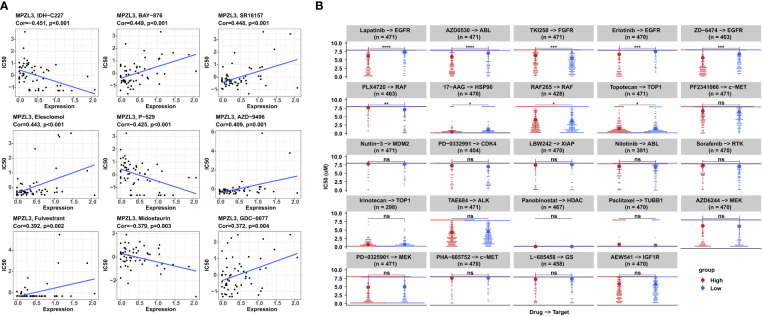
Correlation analysis between MPZL3 expression and drug sensitivity. **(A)** Correlation between MPZL3 and sensitivity to the top 9 anticancer drugs in the CellMiner database. **(B)** Differences in drug sensitivity between the MPZL3 high- and low-expression groups in the GDSC database. MPZL3, Myelin Protein Zero-like 3; GDSC, Genomics of Drug Sensitivity in Cancer. (*p < 0.05, **p < 0.01, ***p < 0.001), ns, no significance.

MPZL3 may participate in the evolution of drug resistance, and thus, we analyzed the correlation between MPZL3 expression and drug sensitivity in the top 9 anticancer drugs from the CellMiner database. The results suggested that high MPZL3 expression could decrease the drug IC50 and increase the drug sensitivity of IDH-C227 (selective IDH1R132H inhibitor), P-529 (TORC1/TORC2 inhibitor), and midostaurin (tyrosine kinase inhibitor) while increasing the drug IC50 and decreasing the drug sensitivity of BAY-876 (selective glucose transporter 1 inhibitor), SR16157 (selective ERα modulator), elesclomol (apoptosis inducer), AZD-9496 (selective estrogen receptor downregulator), fulvestrant (estrogen receptor antagonist) and GDC-0077 (selective PI3Kα inhibitor) (**Figure 8**). The sensitivity of drugs targeting different targets (EGFR, ABL, FGFR, RAF, HSP90, TOP1, c-Met, MDM2, CDK4, XIAP, RTK, HDAC, TUBB1, MEK, GS, IGF1R) was analyzed in the MPZL3 high- and low-expression groups. In contrast, the group with higher MPZL3 expression was more sensitive to lapatinib, erlotinib, and ZD-6474, which specifically target EGFR, more sensitive to AZD0530, which targets ABL, and more sensitive to TKI258, which targets FGFR (**Figure 8B**).

### MPZL3 expression and breast cancer cell proliferation and drug sensitivity

**Figure 9:**
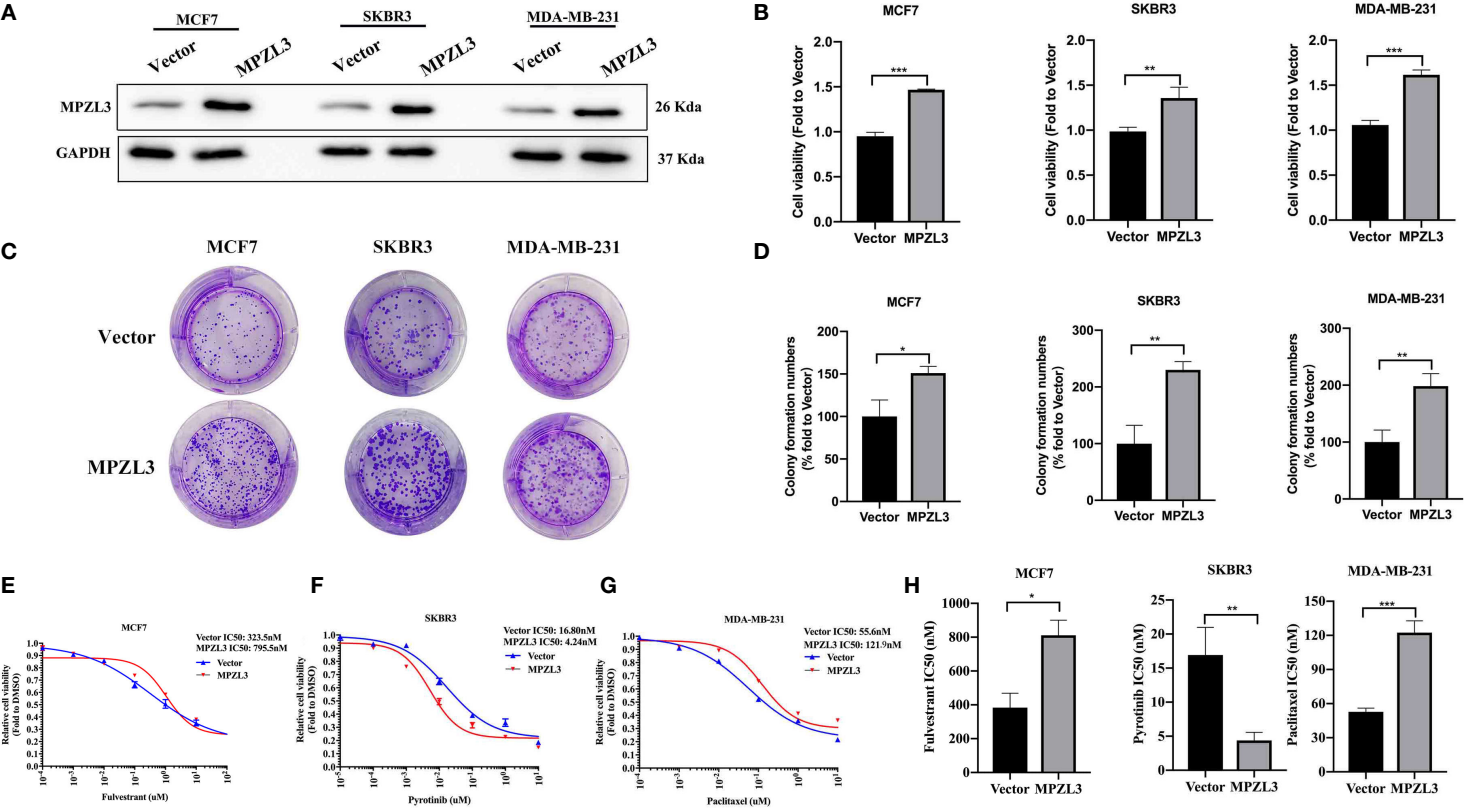
Role of MPZL3 in cell proliferation and drug sensitivity among different breast cancer subtypes. Western blotting **(A)** was applied to detect MPZL3 expression after breast cancer cell lines were transfected with vector control and the MPZL3-overexpression plasmid. The cell proliferation ability **(B)** and colony formation **(C, D)** were assessed after breast cancer cell lines were transfected with vector control and the MPZL3-overexpression plasmid. **(E–H)** The drug sensitivity of fulvestrant, pyrotinib, and paclitaxel was measured in MCF7, SKBR3, and MDA-MB-231 cells after transfection with vector control and the MPZL3-overexpression plasmid. MPZL3, Myelin Protein Zero-like 3. (*p < 0.05, **p < 0.01, ***p < 0.001).

To investigate the role of MPZL3 in breast cancer (BC), we first constructed BC cell lines with stable transduction and high expression of MPZL3. Western blot analysis indicated that MPZL3 was highly expressed after transfection with the MPZL3 plasmid (**Figure 9A**). Then, we detected the differences in proliferation and colony formation between the vector control and the MPZL3 group. The results showed that high MPZL3 expression could promote proliferation and enhance colony formation in MCF7, SKBR3, and MDA-MB-231 cell lines (**Figures 9B–D** **)**. Furthermore, MCF7 cells overexpressing MPZL3 had a higher IC50 value of fulvestrant than MCF7 cells overexpressing the vector control, which indicated that MPZL3 gene overexpression made ER-positive BC cells less sensitive to fulvestrant. Similarly, MDA-MB-231 cells overexpressing the MPZL3 gene were less sensitive to paclitaxel. Conversely, SKBR3 cells overexpressing the MPZL3 gene were more sensitive to pyrotinib (**Figures 9E–H**).

## Discussion

Increasing evidence has provided insights into the tumor immune environment, which aims to identify therapeutic targets for various cancers. Recently, pan-cancer analysis has provided an excellent strategy for revealing certain oncogenes and investigating their mutations, RNA alterations, immune infiltrations, drug susceptibility, and prognostic values in tumors, thus providing insights into novel therapeutic drug development. MPZL3 is a mitochondrially localized membrane protein that exerts a significant role in cell adhesion, antigen binding, skin development, hair growth, etc. The oncogenic role and underlying molecular mechanisms of MPZL3 dysregulation, immune-regulatory function, and prognostic biomarkers of MPZL3 have not been fully elucidated. Because of the potential prognostic value, biological function, and possible drug sensitivity guidance that MPZL3 brings, it is necessary for us to further explore the fundamental mechanism of MPZL3 and its dysregulation across cancers. Under pan-cancer analysis, we successfully identified that MPZL3 acts as a pan-cancer gene and investigated its differential expression, prognostic value, tumor immune infiltration, and drug susceptibility in human cancers. In addition, in breast cancer, we identified that MPZL3 is capable of promoting breast cancer cell proliferation and exerts an essential influence on drug therapeutic options, suggesting that MPZL3 serves as a potential prognostic biomarker for breast cancer.

The present study found that the MPZL3 gene was remarkably highly expressed in ACC, BLCA, BRCA, CESC, CHOL, COAD, ESCA, GBM, KIRP, LAML, LIHC, LUAD, OV, PAAD, PRAD, STAD, TGCT, THCA, UCEC and UCS and expressed at low levels in HNSC, LUSC, and SKCM. Stern YE et al. also demonstrated that MPZL3 mRNA is remarkably overexpressed in MET‐, EGFR‐ and ERBB2‐amplified cancer cell lines and gastric cancer tissues (17). Although the results indicated that MPZL3 is highly expressed in the majority of tumors and can be regarded as an oncogene, a previous study demonstrated that MPZL3 is highly induced in the process of epidermal differentiation and downregulated in cutaneous squamous cell carcinoma (cSCC), which is similar to our finding that MPZL3 is downregulated in SKCM. In a gene expression profile analysis of breast cancer, MPZL3 was one of the significantly downregulated genes in premalignant adjacent tissues compared with the corresponding tumor tissues (18). MPZL3 mRNA has been reported to be highly expressed in radioresistant rectal cancer cell lines (19). All of this evidence implies that MPZL3 can be used as a possible oncogene and promising biomarker for the diagnosis of pan-cancer.

The Kaplan–Meier survival analysis of OS, PFI, DSS, and DFI by TCGA data identified that MPZL3 mRNA expression levels have a prognostic role across cancers. Concerning prognosis, patients with high MPZL3 expression had a relatively worse OS in ACC, BRCA, GBM, LAML, LGG, and PAAD; worse PFI in GBM, LGG, LIHC, and PAAD; worse DSS in ACC, BRCA, GBM, LGG, PAAD, and PRAD; and worse DFI in PAAD. Higher MPZL3 mRNA levels were also linked with better PFI in KIRC and better DSS in THCA. Overall, high MPZL3 expression was linked to poor prognosis across cancers, including breast cancer. A few studies have reported the value of MPZL3 in cancers. For instance, elevated levels of MPZL3 have been demonstrated to be associated with reduced recurrence-free survival (RFS) in STAD and LUSC, which is consistent with our study (20). MPZL3, as one component of a set of gene signatures, could be deemed a novel risk factor and functioned as a prognostic predictor for patients with GBM (21). Thus, previous studies and ours all indicated that MPZL3 mRNA expression might be a reliable diagnostic factor and potentially promising biomarker for pan-cancer diagnosis.

Tumor-associated signaling pathways, immune regulation, cell cycle, apoptosis, chemokine-related pathways, chemokine receptor proteins, etc., are closely associated with MPZL3 expression. KEGG also inferred that MPZL3 expression is related to protein secretion, the Notch signaling pathway, and heme metabolism. The interactions between MPZL3 and cellular proteins or intracellular signaling pathways have yet to be determined in a mammalian system, and the genetic ablation of MPZL3 increases energy expenditure, controls body weight regulation, improves glycemic control and reduces hepatic lipid synthesis (5). A previous study suggested that ROS during epidermal differentiation exert their functions by modulating NOTCH signaling and MPZL3 and FDXR expression (22, 23). Therefore, we speculated that MPZL3 expression exerted different biological functions in different types of cancers.

However, few studies have noted the function of MPZL3 in the immune microenvironment. Polymorphism and mutation analyses of MPZL3 gene expression indicated the possibility that homozygous or compound heterozygous mutations of MPZL3 are related to immune-mediated hereditary hair loss (31). In seborrheic dermatitis-like lesions of MPZL3-knockout mice, IL17 was more highly expressed in γδ T cells to mediate the pathogenesis of seborrheic dermatitis-like skin inflammation (32). The TME contains nonimmune stromal and immune components, and a range of algorithms, such as CIBERSORT, TIMER, ESTIMATE, and MCPcounter, have been successfully applied to evaluate immune and stromal cell infiltration (33). After a series of analyses, it was revealed that MPZL3 was positively correlated with the StromalScore in SKCM, LCG, and STAD, negatively correlated with the ImmuneScore in SKCM, STAD, and LAML, and positively correlated with the ESTIMATEScore in STAD, SKCM, and LGG. A statistically positive correlation was observed between the association of CD8+ T cells, CD4+ T immune cells, B cells, etc., infiltration and MPZL3 expression in BRCA. High expression levels of MPZL3 have been reported to be correlated with the signaling pathway of immune cells. It was indicated by a previous study that expression of MPZL3 in immune cells like dendritic, CD4, and CD8 central memory and effector T cells have supported its potential immune-related role and mutations within the conserved V-type domain of MPZL3 influence immune function, and thus contribute to immune-system deficiencies (17). Furthermore,Mpzl3 knockout is directly or indirectly involved in immune function, and influences CD4, CD8, CD11b, and CD9 immune cell infiltration (34). Therefore, we hypothesized that MPZL3 could mediate immune functions in breast cancer. A better understanding of the complexity and diversity of the immune context of the TME that MPZL3 brings may help to predict and guide immunotherapeutic responsiveness.

MPZL3 was enabled to participate in the evolution of drug resistance, which suggests that MPZL3 can be applied as a target to reverse drug resistance. High MPZL3 expression is associated with increased sensitivity to therapeutic drugs that specifically target EGFR, ABL, and FGFR. To analyze the role of MPZL3 in different subtypes of breast cancer, we first constructed MPZL3-overexpressing cell lines. It was found that MPZL3 can promote proliferation in breast cancer cell lines. Next, we also found that MPZL3 gene overexpression reduces the sensitivity of MCF7 cells to fulvestrant and MDA-MB-231 cells to paclitaxel and increases the sensitivity of SKBR3 cells to pyrotinib. For ER-positive breast cancer, endocrine therapy is a standard therapy. MPZL3, as an oncogene, decreases the drug sensitivity of endocrine and chemotherapy drugs, possibly based on the characteristics of cell promotion and the stimulation of pathways such as PI3K/AKT (35). Previous studies demonstrated that MPZL3 can interact directly with HER3, and the HER3-MPZL3 axis could help explain why Met and EGFR family receptors are vital bypass pathways in models of resistance to EGFR or HER2 inhibition (20). Thus, we speculate that the MPZL3-HER3 axis might be the main reason to explain why MPZL3 can make SKBR3 more sensitive to pyrotinib. This finding suggests that MPZL3 may be a potential therapeutic target for breast cancer.

Conclusively, all of the present studies showed that MPZL3 was upregulated in pan-cancer tissues, and high MPZL3 expression was correlated with worse survival outcomes in pan-cancer. Furthermore, TMB, MSI, MMR, DNA methylation, and RNA modification play a significant role in mediating MPZL3 dysregulation in cancers, and MPZL3 is closely linked to tumor immunity and acts as a suitable target for antitumor immunity therapeutics. Furthermore, drug sensitivity analysis and validation in breast cancer also indicate that MPZL3 might be a potential target for anticancer therapy.

## Data availability statement

The datasets presented in this study can be found in online repositories. The names of the repository/repositories and accession number(s) can be found in the article/**Supplementary material**.

## Author contributions

SKW and WZ conceived and designed the study. HRH and LLQ downloaded and interpreted the data. HRH, LLQ and WZ integrated the figures and written the draft together. SKW and WZ performed the visualization and revised the draft. All authors in the study have contributed to the article and approved the submitted version finally.

## Funding

This study was supported by the National Natural Science Foundation of China (82002773 and 82072897).

## Conflict of interest

The authors declare that the research was conducted in the absence of any commercial or financial relationships that could be construed as a potential conflict of interest.

## Publisher’s note

All claims expressed in this article are solely those of the authors and do not necessarily represent those of their affiliated organizations, or those of the publisher, the editors and the reviewers. Any product that may be evaluated in this article, or claim that may be made by its manufacturer, is not guaranteed or endorsed by the publisher.

## Supplementary material

Supplementary Figure 1 Survival curves of OS in ACC, GBM, LAML, LGG, and PAAD patients from TCGA database. OS, overall survival; ACC, adrenocortical carcinoma; GBM, glioblastoma multiforme; LAML, acute myeloid leukemia; LGG, low-grade glioma; PAAD, pancreatic adenocarcinoma; TCGA, The Cancer Genome Atlas.

Supplementary Figure 2 Survival curves of PFI in GBM, KIRC, LGG, LIHC, and PAAD patients from TCGA database. PFI, progression-free interval; GBM, glioblastoma multiforme; KIRC, kidney renal clear cell carcinoma; LGG, low-grade glioma; LIHC, liver hepatocellular carcinoma; PAAD, pancreatic adenocarcinoma; TCGA, The Cancer Genome Atlas.

Supplementary Figure 3 Survival curves of DSS in ACC, GBM, LGG, PAAD PRAD and THCA patients from the TCGA database. DSS, disease-specific survival; ACC, adrenocortical carcinoma; GBM, glioblastoma multiforme; LGG, low-grade glioma; PAAD, pancreatic adenocarcinoma; PRAD, prostate adenocarcinoma; TCGA, The Cancer Genome Atlas.

Supplementary Figure 4 Survival curves of DFI in PAAD patients from TCGA database. DFI, disease-free survival; PAAD, pancreatic adenocarcinoma; TCGA, The Cancer Genome Atlas.

Supplementary Figure 5 Associations of MPZL3 expression with TMB and MSI. **(A)** The radar map illustrates the correlation between MPZL3 expression and TMB. The value in black reveals the range, and the curve in blue reveals the correlation coefficient. **(B)** The radar map illustrates the correlation between MPZL3 expression and MSI. The value in black reveals the range, and the curve in red reveals the correlation coefficient. MPZL3, myelin protein zero-like 3; TMB, tumor mutational burden; MSI, microsatellite instability.

Supplementary Figure 6 Correlation of MPZL3 somatic copy number alterations with immune infiltration levels in different types of BRCA, including Basel, luminal, and HER2. Box plots present the distributions of different immune subsets on the basis of each copy number status. MPZL3, myelin protein zero-like 3; BRCA, breast invasive carcinoma; HER2, human epidermal growth factor receptor 2. (*p < 0.05, **p < 0.01, ***p < 0.001).

Supplementary Figure 7 Correlation of MPZL3 expression with ImmuneScore in various cancers. MPZL3, Myelin Protein Zero-like 3.

Supplementary Figure 8 Correlation of MPZL3 expression with ImmuneScore. (B) Correlation of MPZL3 expression with StromalScore. MPZL3, Myelin Protein Zero-like 3.

Supplementary Figure 9 Correlation of MPZL3 expression with ESTIMATEScore. MPZL3, Myelin Protein Zero-like 3.

Supplementary Figure 10 Correlation of MPZL3 expression with the Timer Database core in BRCA. MPZL3, myelin protein zero-like 3; BRCA, breast invasive carcinoma.

Supplementary Figure 11 GSEA enrichment analysis for samples with high or low MPZL3 mRNA expression. **(A)** Gene sets enriched in KEGG by the samples with high MPZL3 expression levels. **(B)** Gene sets enriched in KEGG by the samples with low MPZL3 expression levels. **(C)** Enriched gene sets in the HALLMARK term by samples with high MPZL3 expression levels. **(D)** Enriched gene sets in the HALLMARK term by samples with low MPZL3 expression levels. GESA, gene set enrichment analysis; MPZL3, myelin protein zero-like 3; KEGG, Kyoto Encyclopedia of Genes and Genomes; MPZL3, myelin protein zero-like 3.
