# Supplementary figures and images for: A systemic pan-cancer analysis of MPZL3 as a potential prognostic biomarker and its correlation with immune infiltration and drug sensitivity in breast cancer
Source: Front Oncol. 2022 Jul 29;12:901728. doi: 10.3389/fonc.2022.901728 (PMC9372439; doi:10.3389/fonc.2022.901728)

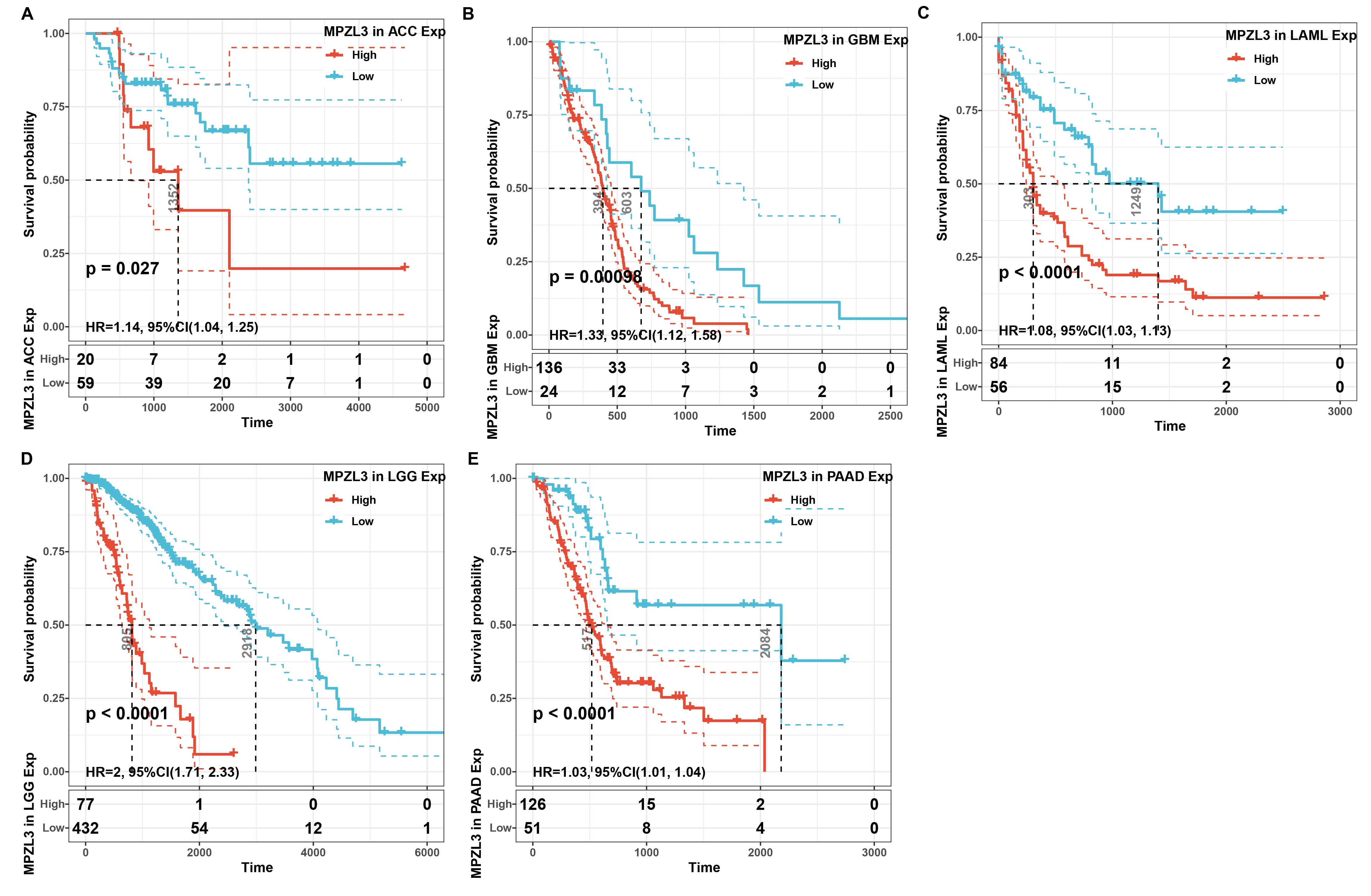

Supplement: Supplementary Figure 1 — Survival curves of OS in ACC, GBM, LAML, LGG, and PAAD patients from TCGA database. OS, overall survival; ACC, adrenocortical carcinoma; GBM, glioblastoma multiforme; LAML, acute myeloid leukemia; LGG, low-grade glioma; PAAD, pancreatic adenocarcinoma; TCGA, The Cancer Genome Atlas. [file Image_1.jpeg]

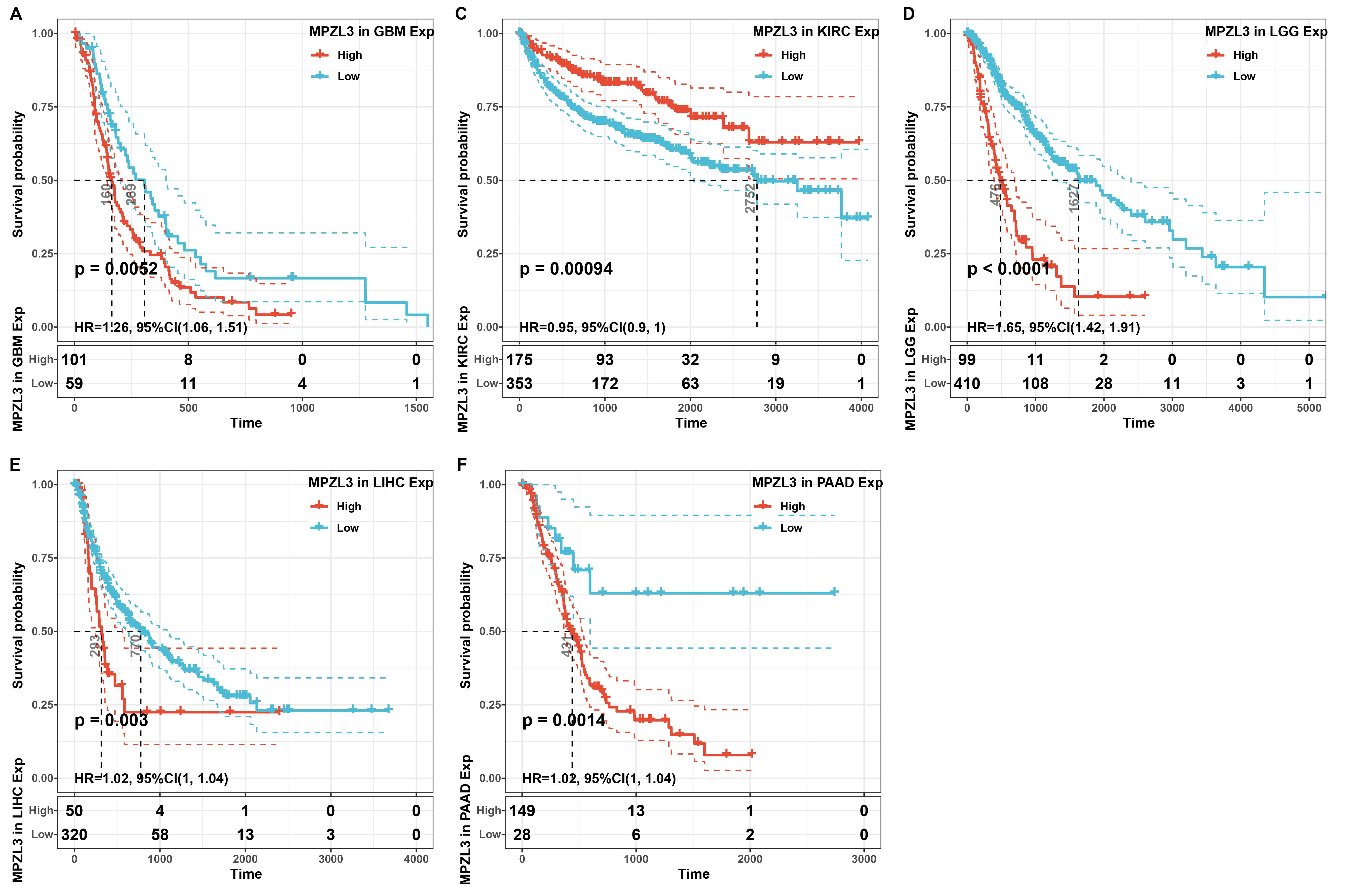

Supplement: Supplementary Figure 2 — Survival curves of PFI in GBM, KIRC, LGG, LIHC, and PAAD patients from TCGA database. PFI, progression-free interval; GBM, glioblastoma multiforme; KIRC, kidney renal clear cell carcinoma; LGG, low-grade glioma; LIHC, liver hepatocellular carcinoma; PAAD, pancreatic adenocarcinoma; TCGA, The Cancer Genome Atlas. [file Image_2.jpeg]

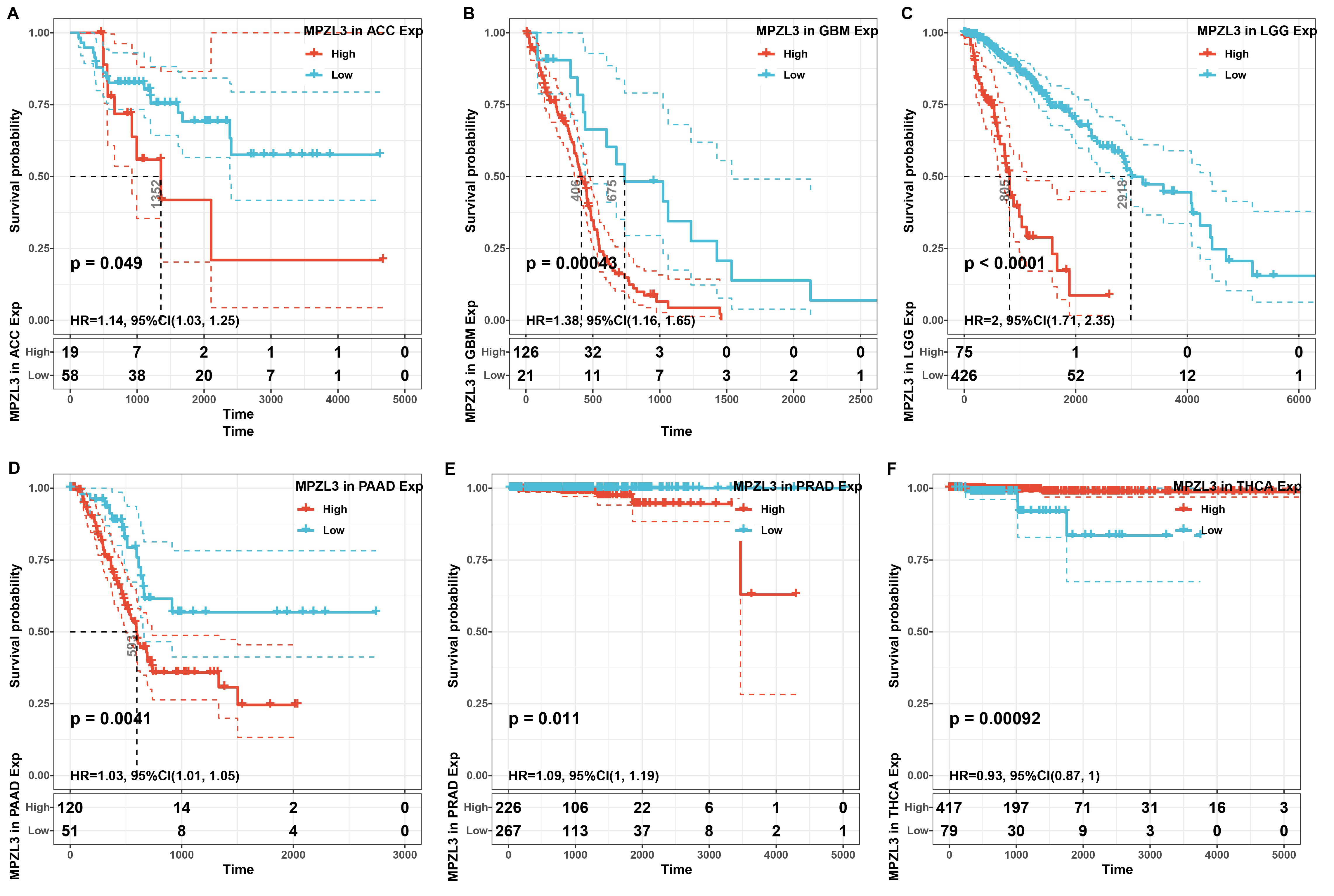

Supplement: Supplementary Figure 3 — Survival curves of DSS in ACC, GBM, LGG, PAAD PRAD and THCA patients from the TCGA database. DSS, disease-specific survival; ACC, adrenocortical carcinoma; GBM, glioblastoma multiforme; LGG, low-grade glioma; PAAD, pancreatic adenocarcinoma; PRAD, prostate adenocarcinoma; TCGA, The Cancer Genome Atlas. [file Image_3.jpeg]

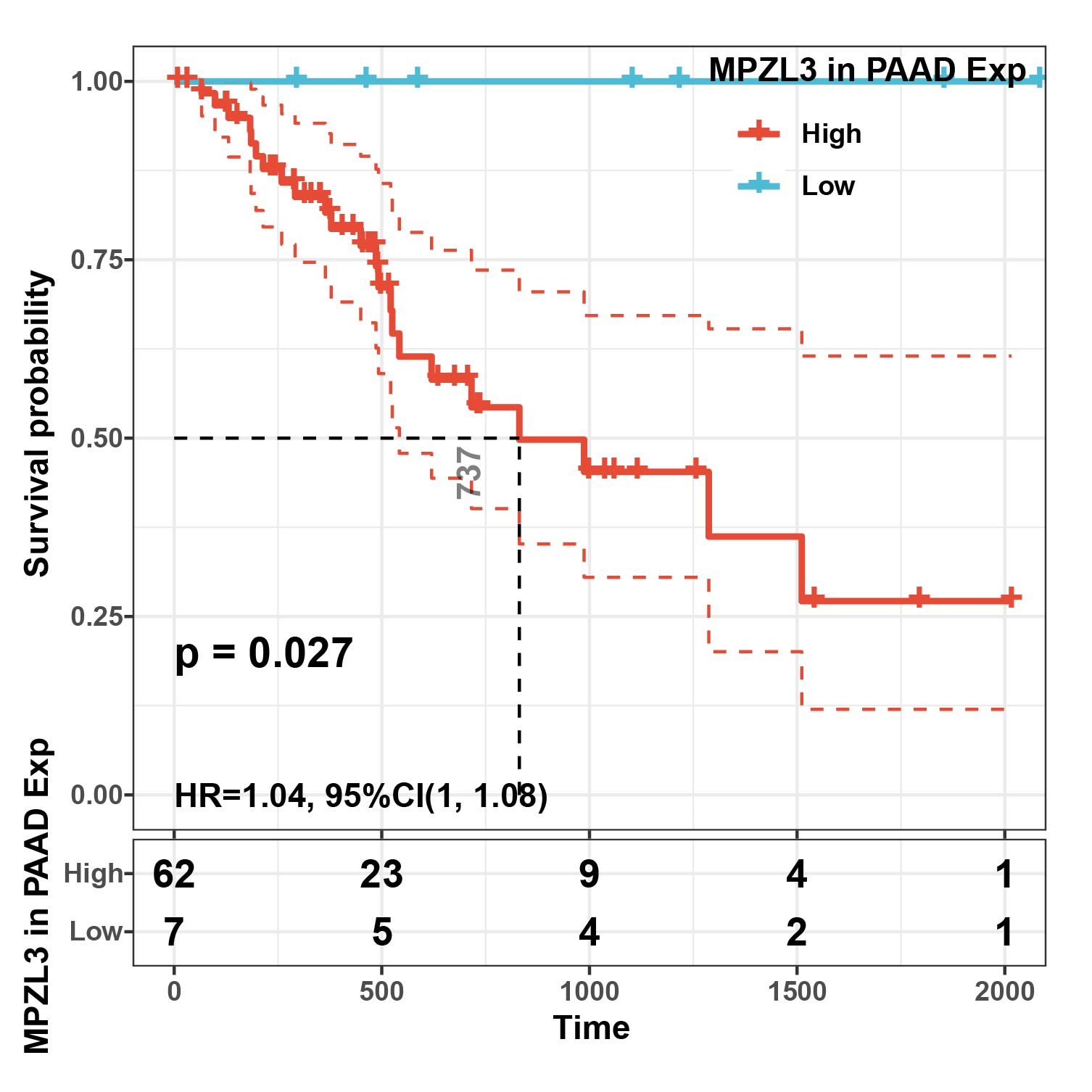

Supplement: Supplementary Figure 4 — Survival curves of DFI in PAAD patients from TCGA database. DFI, disease-free survival; PAAD, pancreatic adenocarcinoma; TCGA, The Cancer Genome Atlas. [file Image_4.jpeg]

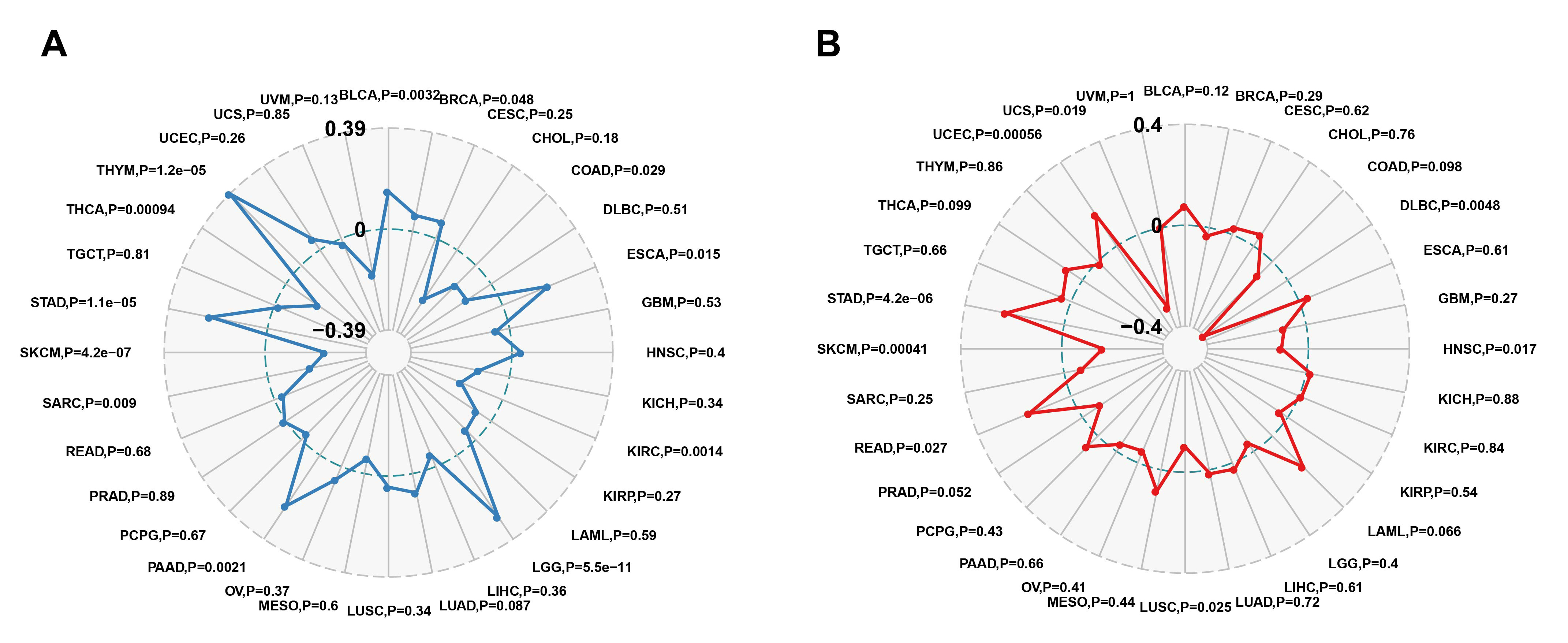

Supplement: Supplementary Figure 5 — Associations of MPZL3 expression with TMB and MSI. (A) The radar map illustrates the correlation between MPZL3 expression and TMB. The value in black reveals the range, and the curve in blue reveals the correlation coefficient. (B) The radar map illustrates the correlation between MPZL3 expression and MSI. The value in black reveals the range, and the curve in red reveals the correlation coefficient. MPZL3, myelin protein zero-like 3; TMB, tumor mutational burden; MSI, microsatellite instability. [file Image_5.jpeg]

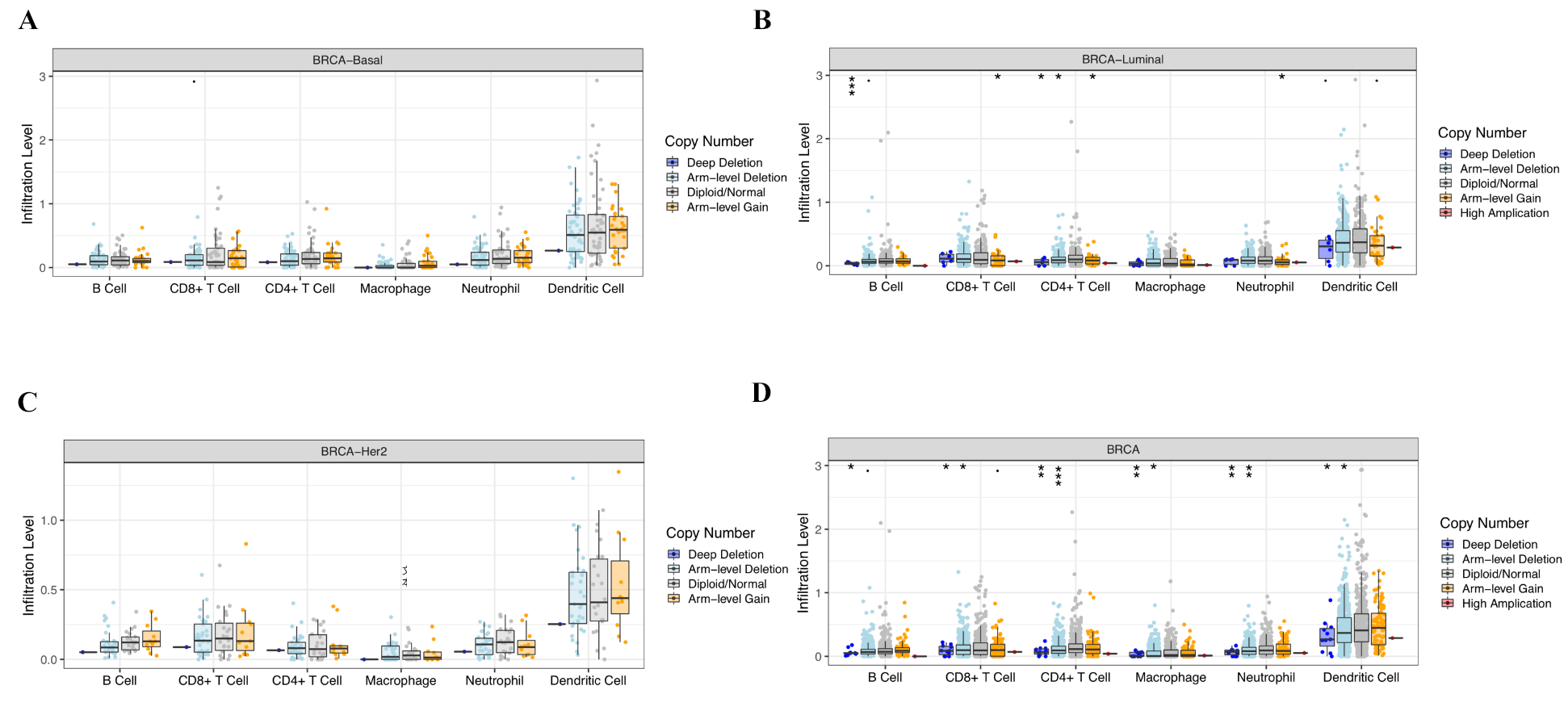

Supplement: Supplementary Figure 6 — Correlation of MPZL3 somatic copy number alterations with immune infiltration levels in different types of BRCA, including Basel, luminal, and HER2. Box plots present the distributions of different immune subsets on the basis of each copy number status. MPZL3, myelin protein zero-like 3; BRCA, breast invasive carcinoma; HER2, human epidermal growth factor receptor 2. (*p < 0.05, **p < 0.01, ***p < 0.001). [file Image_6.tif]

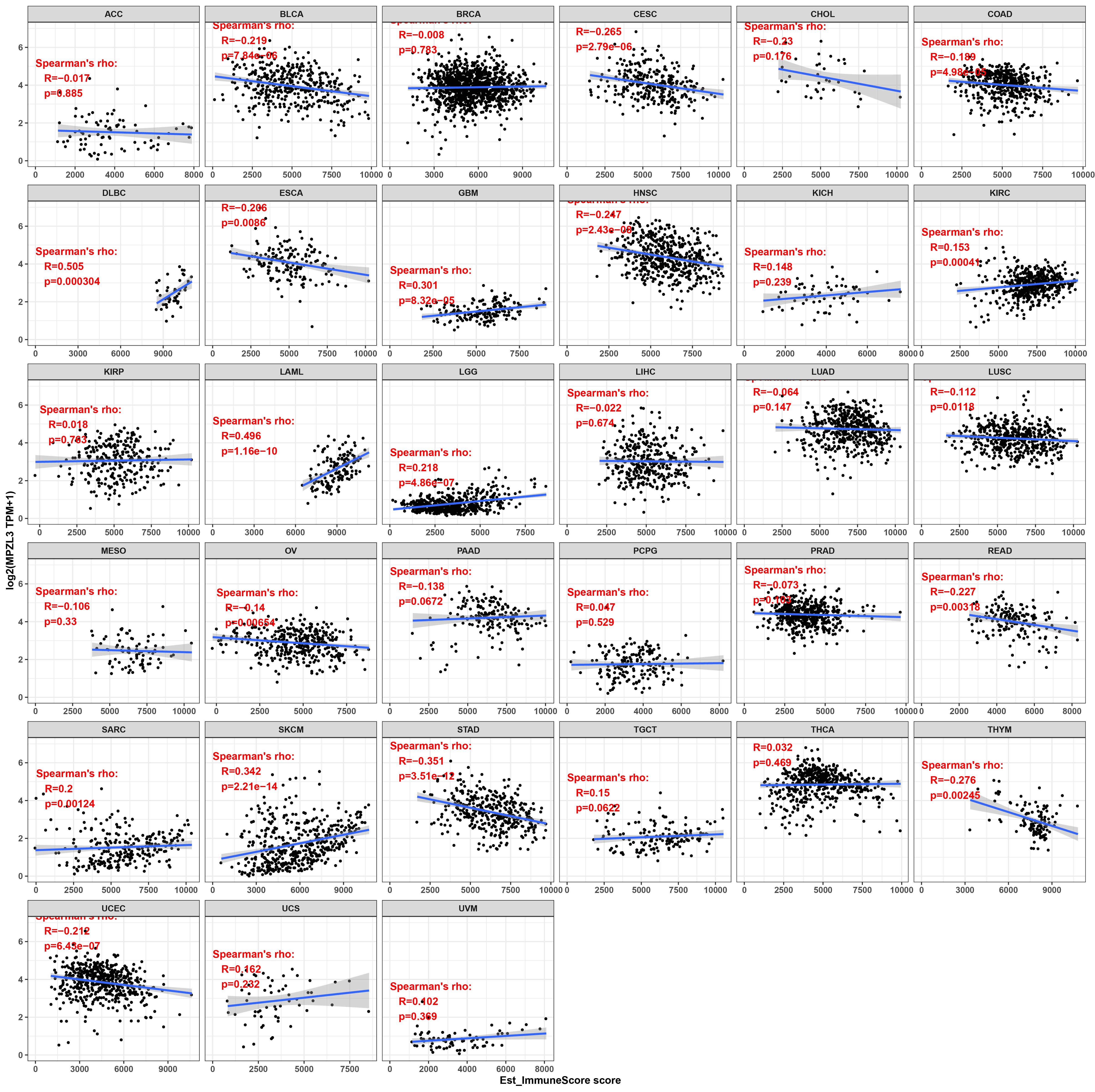

Supplement: Supplementary Figure 7 — Correlation of MPZL3 expression with ImmuneScore in various cancers. MPZL3, Myelin Protein Zero-like 3. [file Image_7.jpeg]

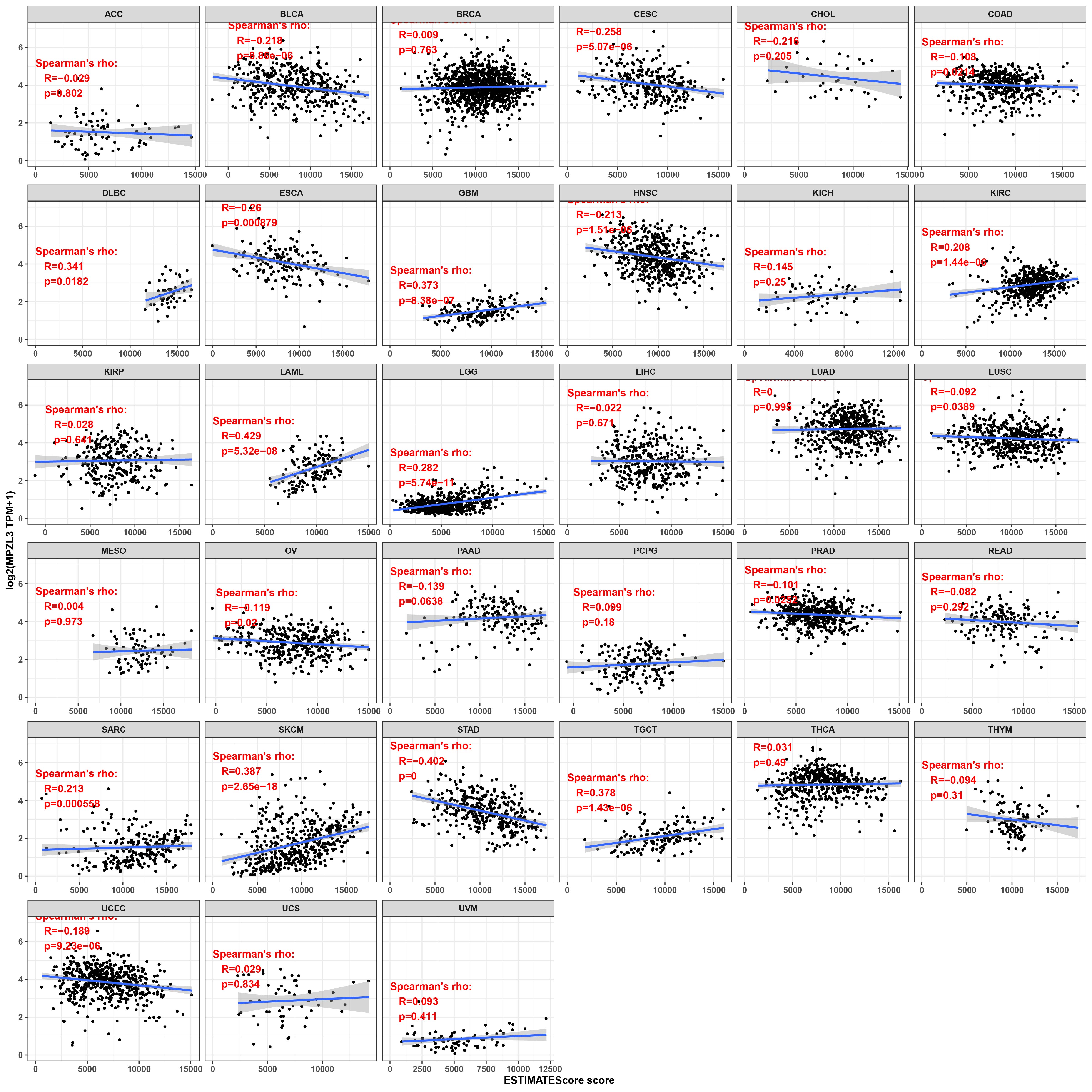

Supplement: Supplementary Figure 8 — Correlation of MPZL3 expression with ImmuneScore. (B) Correlation of MPZL3 expression with StromalScore. MPZL3, Myelin Protein Zero-like 3. [file Image_8.jpeg]

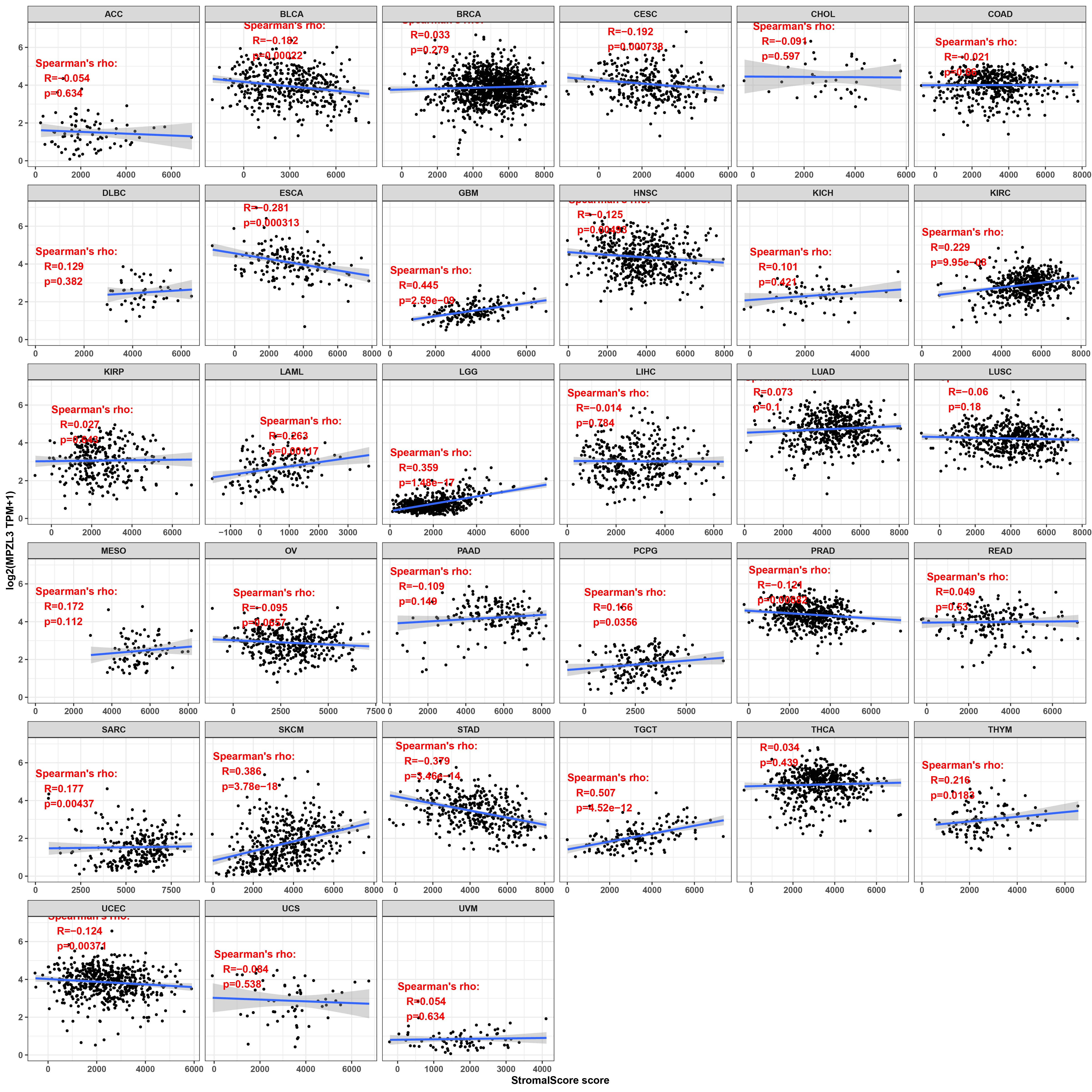

Supplement: Supplementary Figure 9 — Correlation of MPZL3 expression with ESTIMATEScore. MPZL3, Myelin Protein Zero-like 3. [file Image_9.jpeg]

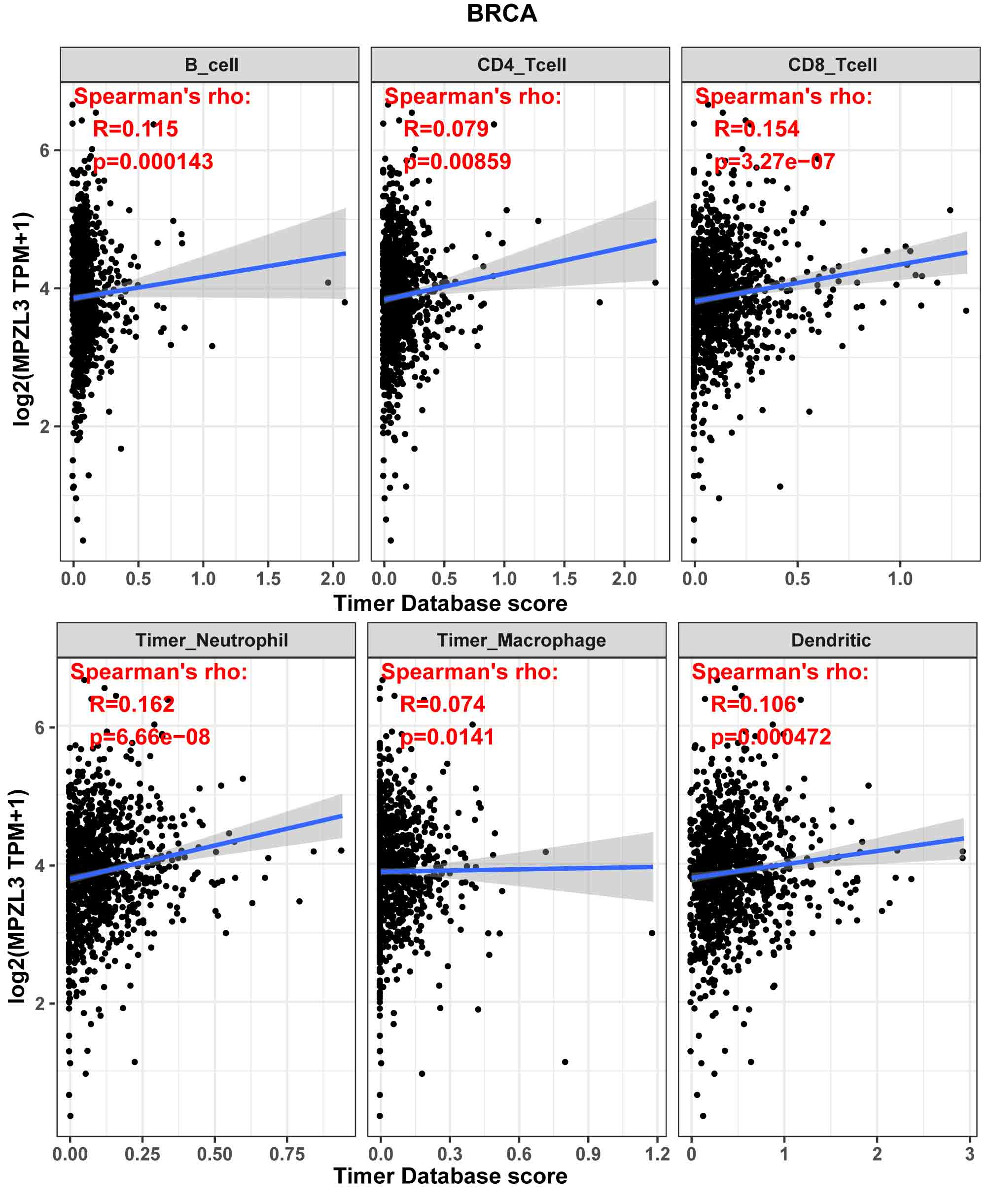

Supplement: Supplementary Figure 10 — Correlation of MPZL3 expression with the Timer Database core in BRCA. MPZL3, myelin protein zero-like 3; BRCA, breast invasive carcinoma. [file Image_10.jpeg]

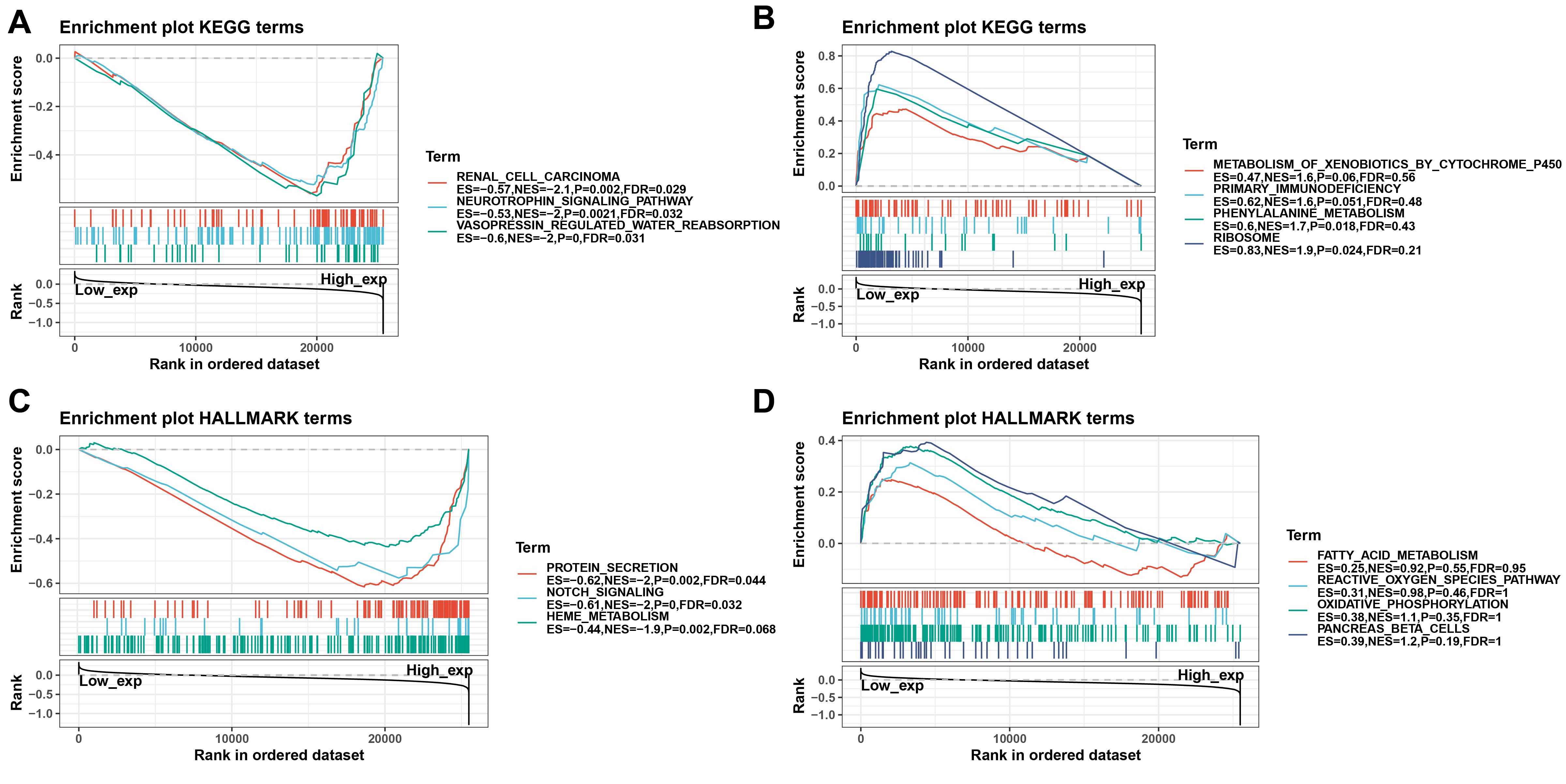

Supplement: Supplementary Figure 11 — GSEA enrichment analysis for samples with high or low MPZL3 mRNA expression. (A) Gene sets enriched in KEGG by the samples with high MPZL3 expression levels. (B) Gene sets enriched in KEGG by the samples with low MPZL3 expression levels. (C) Enriched gene sets in the HALLMARK term by samples with high MPZL3 expression levels. (D) Enriched gene sets in the HALLMARK term by samples with low MPZL3 expression levels. GESA, gene set enrichment analysis; MPZL3, myelin protein zero-like 3; KEGG, Kyoto Encyclopedia of Genes and Genomes; MPZL3, myelin protein zero-like 3. [file Image_11.jpeg]
